# Pregnancy Galectinology: Insights Into a Complex Network of Glycan Binding Proteins

**DOI:** 10.3389/fimmu.2019.01166

**Published:** 2019-05-29

**Authors:** Sandra M. Blois, Gabriela Dveksler, Gerardo R. Vasta, Nancy Freitag, Véronique Blanchard, Gabriela Barrientos

**Affiliations:** ^1^Reproductive Medicine Research Group, Division of General Internal and Psychosomatic Medicine, Berlin Institute of Health, Charité – Universitätsmedizin Berlin, Corporate Member of Freie Universität Berlin, Humboldt-Universität zu Berlin, Berlin, Germany; ^2^Experimental and Clinical Research Center, a Cooperation Between the Max Delbrück Center for Molecular Medicine in the Helmholtz Association, and Charité - Universitätsmedizin Berlin, Berlin, Germany; ^3^Department of Pathology, Uniformed Services University of the Health Sciences, Bethesda, MD, United States; ^4^Department of Microbiology and Immunology, Institute of Marine and Environmental Technology, University of Maryland School of Medicine, UMB, Baltimore, MD, United States; ^5^Berlin Institute of Health, Institute of Laboratory Medicine, Clinical Chemistry and Pathobiochemistry, Charité – Universitätsmedizin Berlin, Corporate Member of Freie Universität Berlin, Humboldt-Universität zu Berlin, Berlin, Germany; ^6^Laboratory of Experimental Medicine, Hospital Alemán, School of Medicine, University of Buenos Aires, CONICET, Buenos Aires, Argentina

**Keywords:** galectins, pregnancy, placentation, glycans, preeclampsia

## Abstract

Galectins are a phylogenetically conserved family of soluble β-galactoside binding proteins, consisting of 15 different types, each with a specific function. Galectins contribute to placentation by regulating trophoblast development, migration, and invasion during early pregnancy. In addition, galectins are critical players regulating maternal immune tolerance to the embedded embryo. Recently, the role of galectins in angiogenesis during decidualization and in placenta formation has gained attention. Altered expression of galectins is associated with abnormal pregnancies and infertility. This review focuses on the role of galectins in pregnancy-associated processes and discusses the relevance of galectin-glycan interactions as potential therapeutic targets in pregnancy disorders.

## Introduction

During pregnancy, a delicately regulated interplay of endocrine, immune and metabolic processes is established in order to sustain offspring development. The coordination of a series of simultaneous events occurring at both sides of the maternal-fetal interface, including multiple signaling pathways driving cell growth and differentiation, vascular development, and immune regulation, is critical for a successful pregnancy outcome. At the maternal site, complex immunoregulatory mechanisms support active tolerance of fetal alloantigens while also remaining competent to elicit an effective response toward pathogenic insults (1). Throughout pregnancy the uterine vascular bed experiences dramatical changes with extensive remodeling of existing vessels and formation of new networks through the process of angiogenesis (2), allowing for the proper delivery of oxygen and nutrients to the developing fetus. In parallel, at the fetal site, the process of placentation relies on a complex interaction between invasive trophoblasts and maternal immune cells involving developmentally regulated periods of branching angiogenesis, non-branching angiogenesis, trophoblast differentiation and syncytium formation. Disruption of this normal pattern of placental development will directly impact placental function, with well-recognized consequences leading to suboptimal pregnancy outcomes (3).

The placenta sustains pregnancy by providing an immunological barrier between the mother and fetus, mediating the transfer of gases, nutrients and water and secreting a variety of hormones, cytokines, and signaling factors. As the active interface mediating maternal-fetal communication, the placenta plays a key role in sensing and modulating perturbations in the maternal environment and transmitting these stimuli to the developing fetus, with potential consequences in long-term offspring health. Indeed, it is now well-recognized than an adverse intrauterine environment during early development can modify disease predisposition in adult life as stated in the so-called “developmental origins of health and disease” or “fetal programming” paradigm. From the time of pioneer studies correlating birth weight and altered fetal growth with predisposition to chronic conditions as cardiovascular disease and diabetes (4), accumulating experimental evidence has contributed to the identification of multiple maternal constitutional and life style factors that can impact long-term individual health as well as the mechanisms involved in the transmission of these programming stimuli across the placenta (5–7).

Among the multiple mediators involved in pregnancy orchestration, the galectin family of β-galactoside binding proteins elicits great interest in the reproductive medicine field due to their unique ability to modulate diverse developmental processes and their potential use as biomarkers for gestational disorders. In this review we discuss the current knowledge on the role of galectins in supporting maternal adaptations to pregnancy and placental development, the impact of their dysregulation for development of disease and the potential application of galectinome profiling studies for diagnostic and therapeutic interventions in adverse pregnancy outcomes.

## General Aspects of Galectins

Complex carbohydrates on the cell surface and the extracellular matrix (ECM) encode abundant structural information that when decoded by specific carbohydrate-binding proteins (lectins) modulates interactions between cells, or cells and the ECM (8, 9). Based on their structural folds and canonical sequence motifs in the carbohydrate recognition domain (CRD), lectins have been organized into several families that include galectins (formerly S-type lectins), C-type, F-type, X-type, R-type, P-type, and several other families [Reviewed in (10)]. The taxonomic distribution of selected lectin families and their structural analysis have yielded critical information about their functional aspects and evolutionary history. While F- and C-type lectins—are largely heterogeneous and evolutionary diversified lectin families (11, 12), from a structural standpoint, galectins are relatively conserved (13, 14). Galectins are non-glycosylated soluble proteins characterized by a unique sequence motif in their CRDs and affinity for β-galactosides (13). Most galectins show preference for N-acetylated disaccharides such as N-acetyl-lactosamine (LacNAc; Galβ1,4GlcNAc) and related structures, whereas others have preference for blood group oligosaccharides (13–15).

**Figure 1:**
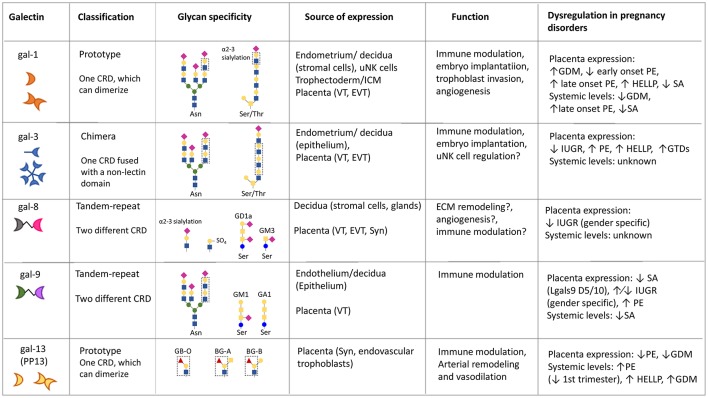
Summary of galectins' glycan specificity, functions and sources of expression in mammalian pregnancies. Question marks (?) denote putative functions (not yet experimentally confirmed in the context of pregnancy). BG, blood group; uNK, uterine natural killer; ICM, inner cell mass; VT, villous cytotrophoblast; EVT, extravillous trophoblast; Syn, syncytiotrophoblast; GDM, gestational *diabetes mellitus;* SA, spontaneous abortion; PE, preeclampsia; HELLP, hemolysis elevated liver enzymes and low platelet syndrome; GTD, gestational trophoblastic disease; IUGR, intrauterine growth restriction. ↑ and ↓ denote increased/peak and decreased expression, respectively. 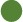 Mannose 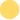 Galactose 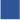 N-acetylglucosamine 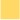 N-acetylgalactosamine 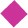 Sialic Acid 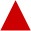 Fucose.

Galectins are synthesized in the cytoplasm, and can be translocated into the nucleus where they can form part of the spliceosome (16, 17). Galectins can also be secreted to the extracellular space by non-classical mechanisms as they lack a typical signal peptide possibly by direct transport across the plasma membrane (18). Once secreted, galectins bind to carbohydrate ligands in the ECM and the cell surface, namely glycans that display LacNAc and polylactosamine chains [(Galβ1, 4GlcNAc)n] (13) (Figure 1). These include laminin and fibronectin, mucins, lysosome-associated membrane proteins, and numerous cell surface glycoproteins (19–22). Among the latter, galectins bind with high affinity to glycosylated cell surface signaling molecules such as α and β integrins (21) and the signaling mucin MUC1 (22). Integrins are the predominant laminin and fibronectin-binding proteins expressed on differentiating cells and represent important ligands for galectins involved in cell adhesion, motility and differentiation (21–24). For some galectins, the immediate binding to ligands in the oxidative extracellular environment is key to the stability of their carbohydrate-binding activity (25). The crystal structure of galectin-1 (Lgals1) revealed six key cysteine residues, some of which are located on the surface of the molecule on the face opposite to the CRD and are potentially susceptible to oxidation (26–28). Under non-reducing conditions, intramolecular disulfide bridges are formed resulting in conformational changes that preclude Lgals1 from forming a dimer (29). A critical interplay takes place between the oxidation state of cysteine sulfhydryl groups and the ligand binding and dimerization equilibrium, suggesting that specific binding to glycan ligands enhances dimerization and reduces sensitivity to oxidative inactivation (25). In addition, extracellular galectins can also recognize exogenous ligands, such as glycans on the surface of viruses, bacterial pathogens and parasites (30–33), a hallmark of other lectin types, such as C-type lectins (11). Furthermore, galectin secretion into the extracellular space upon stress has been proposed to constitute non-infectious “danger signals” that can initiate or exacerbate inflammatory responses (34).

## Structural Aspects of Galectins

Galectins are characterized by their extensive taxonomic distribution and striking evolutionary conservation of primary structures, gene organization, and structural fold (11). The identification of galectin-like proteins in the fungus *Coprinopsis cinerea* (35) and in the sponge *Geodia cydonium* (36) revealed structural conservation of galectins in eukaryotic evolution. Furthermore, proteins sharing the galectin structural fold identified in the protozoan parasite *Toxoplasma gondii* (37, 38) and in rotaviruses (39–41) suggest either early emergence of the galectin fold or horizontal transfer from the vertebrate hosts, respectively. In general, galectin polypeptide subunits exhibit a relatively simple domain organization, housing one, two, or four galectin CRDs (11).

Although galectins have been evolutionarily conserved (42), the galectin repertoire in any given mammalian species is constituted by multiple galectin types, subtypes, and isoforms (13). Based on the CRD organization of the polypeptide monomer, mammalian galectins (and by extension, galectins in all vertebrate taxa) have been classified in three major types: “proto,” “chimera,” and “tandem-repeat” (TR) types (43) (Figure 1). Proto-type galectins contain one CRD per subunit and can form concentration-dependent non-covalently linked homodimers. Dimerization of proto-type galectins is key to their function in mediating cell-cell or cell-ECM interactions (44, 45). Two Lgals1 monomers interact via amino acid residues from a hydrophobic core that establish a dimerization equilibrium with a Kd of 7 μM (16). Both proto- and TR-type galectins comprise several distinct subtypes, all numbered in the order of their discovery, while chimera galectins include a single subtype (13). Lgals1,−2,−5,−7,−10,−11,−13,−14, and−15, are included in the proto-type. Chimera-type galectins, represented by Lgals3, have a C-terminal CRD and a proline- and glycine-rich N-terminal “tail.” Ligand-driven interactions of Lgals3 subunits via the N-terminal domain mediate their oligomerization into trimers and pentamers (46). TR galectins display two similar—albeit not identical—CRDs connected by a functional linker peptide (47), and comprise the Lgals4, −6, −8, −9, and −12 subtypes. Galectin subtypes may be expressed as multiple isoforms in a single cell type or as tissue-specific variants generated by alternative splicing (13, 48), positive selection, and amino acid replacements in carbohydrate-recognition domains (49). Among the proto, chimera and TR galectin types, several subtypes, that include human Lgals1, Lgals2, Lgals3, Lgals9, and three galectins that cluster in the human chromosome 19 [Lgals13 (pp. 13), −14, and −16], have been recently investigated with regards to their potential roles in fertilization, embryo implantation, placentation, and the various stages of normal and pathological pregnancy (49–52).

The structural fold of Lgals1 and the amino acid residues of the CRD that directly or indirectly—via water molecules—interact with the hydroxyl groups on the carbohydrate ligands have been identified by the resolution of the crystal structure of the Lgals1/LacNAc complex (26, 27, 53). The Lgals1 subunit is a β-sandwich consisting of a 135 amino acid-long polypeptide that folds into two antiparallel β-sheets of five and six strands each (S1–S6 and F1–F5). This globular structure contains one carbohydrate binding cleft formed by three continuous concave strands (S4–S6) that includes all amino acid residues that interact with LacNAc and are responsible for the carbohydrate specificity of Lgals1: histidine 44, asparagine 46, arginine 48, histidine 52, asparagine 61, tryptophan 68, glutamic acid 71, and arginine 73 (27). Tryptophan 68 establishes a hydrophobic stacking interaction with the non-reducing terminal galactose ring. Additional water-mediated interactions between His52, Asp54, and Arg73 in the Lgals1 CRD with the nitrogen of the NAc group rationalize the higher affinity for LacNAc over lactose. The rigorous assessment of the galectins' carbohydrate-binding affinity has been enabled by biophysical approaches, such as microcalorimetry measurements and surface plasmon resonance analysis. For example, the dissociation constants of bovine Lgals1 for Lac, LacNAc, and thiodigalactoside measured by microcalorimetry were in the range of 10^−5^ M, with two binding sites per Lgals1 dimer (54). The overall structure of the Lgals3 CRD is very similar to the Lgals1 CRD although in the former the carbohydrate-binding site is shaped as a cleft open at both ends, exposing the GlcNAc of the LacNAc to the solvent (55). This extended binding site in Lgals3 results in increased affinity for polylactosamines and for ABH blood group oligosaccharides [Fucα1, 2; GalNAcα1,3(Fucα1,2); and Galα1,3(Fucα1,2)] (55). The structures of the individual N- and C-terminal CRDs of TR galectins, such as galectins-4,- 8, and−9, have been resolved by either crystallization or NMR spectroscopy. Results have shown that the two CRDs in the same galectin molecule are structurally similar but exhibit either different affinities for the same ligand such as observed in Lgals4, or different fold and specificities altogether, such as reported for Lgals8 (56–58).

Based on analysis of the galectin primary structure and intron-exon position in various vertebrate species it has been proposed that along the vertebrate lineages leading to mammals, galectins evolved by duplication of a primordial single CRD galectin gene that produced a bi-CRD gene, with the N- and C-terminal CRDs later diverging into two subtypes (F4-CRD and F3-CRD) of distinct exon-intron organization. Single-CRD galectins display the F3- (e.g., Lgals1, −2, −3, −5) or F4- (e.g., Lgals7, −10, −13, −14) subtypes, while TR galectins display both F4 and F3 subtypes (Lgals4, −6, −8, −9, and −12) (13, 42). In invertebrate species, galectins exhibit one, two, or four tandem-arrayed CRDs (59–61). In those invertebrate galectins that carry multiple CRDs, these are structurally similar but not identical, suggesting that they differ in their fine carbohydrate specificity (61). How the multiple CRD galectins from invertebrates relate to the vertebrate TR galectins remains to be fully understood, but a preliminary phylogenetic analysis revealed that individual CRDs of a four-CRD galectin clusters with the mammalian single CRD galectins rather with the TR galectins, suggesting that this gene is the product of two consecutive duplications of a single-CRD galectin gene (61).

## Functional Aspects of Galectins

As discussed above, TR galectins display two CRDs in a single polypeptide, that can interact with and cross-link multivalent ligands, either soluble glycoproteins or glycolipids, or ECM and complex glycans on the cell surface. Although proto- and chimera-type galectin subunits possess a single CRD, they can organize as oligomeric structures that also bind multivalent ligands with increased avidity (44, 45). The density of the cell surface glycans and their scaffolding (as glycoproteins, glycolipids or polysaccharides) modulates affinity of the CRD-ligand interaction via negative co-operativity (45), and can lead to ligand cross-linking, and formation of lattices that cluster these ligands into lipid raft microdomains (44). These interactions can promote reorganization or association of cell surface components, regulate turnover of endocytic receptors, activate or attenuate signaling pathways, and in turn, modulate cell function (44). Further, because galectin types and subtypes exhibit notable differences in carbohydrate specificity and affinity and bind a broad range of glycans that display the requisite topologies, the galectin repertoire displays considerable diversity in recognition properties that together with their distinct and unique tissue distribution and local concentrations, supports extensive functional diversification (13, 30). Thus, the biological function of a particular galectin may vary among cells, tissues and fluids, depending on their concentration, the availability and multivalent presentation of suitable carbohydrate ligands, and the redox properties of any particular intra- or extracellular microenvironment (30).

## Early Development and Tissue Regeneration

The initial description in the early “80s of developmentally-regulated galectins in chicken muscle suggested that their biological roles were related to embryogenesis and early development. Further, the finding that chicken galectins preferentially recognized the abundant polylactosamines present on the myoblast surface and the ECM, suggested that galectins mediate myoblast fusion [reviewed in (14)]. Later studies suggested roles of murine Lgals1 and Lgals3 in notochord development and somitogenesis, and in skeletal muscle and central nervous system development (62, 63). In recent years, the increasing availability of null mice for selected galectins enabled their developmental phenotypic analysis. Although the phenotypes identified have been in some cases rather subtle, which hindered a rigorous assignment of the galectins” biological roles, the use of galectin deficient models and tissue-specific knockouts is one of the most complete available tools for the analysis of the biological role of galectins. In addition, rodents express a complex galectin repertoire; this was attributed to functional redundancy of the multiple galectin types and subtypes. However, as the binding properties and natural ligands of each galectin have been rigorously characterized in recent years, it has become clear that this is not the case, and their unique biological roles are being elucidated in increasing detail. In the past few years, Drosophila, *C. elegans*, and zebrafish (*Danio rerio*) have become useful model systems to address the biological roles of galectins (64–68). For example, antisense knockdown approaches in zebrafish embryos for a Lgals1 isoform (Drgal1-L2) revealed a key role in differentiation and development of the myotome (69). The zebrafish model was also useful to assess the roles of galectins in tissue repair and regeneration (53, 70). Experimentally light-induced retinal injury in adult zebrafish was used in combination with an antisense knockdown approach to demonstrate that photoreceptor cell death upregulates expression and secretion of DrGal1-L2 by stem cells and neuronal progenitors in the Müller glia, and selectively regulates the regeneration of rod photoreceptors (70).

## Genome Organization of Galectin Family Members

While galectins are usually grouped based on their architecture, a potential relationship between gene location and function has been investigated (42, 50). Genes encoding Lgals1 and Lgals2, named *LGALS* in humans and *Lgals* in mice and other chordates, map to syntenic regions of chromosome 22 and chromosome 15, in humans and mice, respectively. Studies of the promoter sequences and expression of murine *Lgal*s*1*, -*2* and -*7* revealed significant differences in the proximal promoter regions for putative transcription factor binding sites in these genes, which is believed to correlate with the ubiquitous gene expression of *Lgals*1 and a more restricted expression of *Lgals*2 and *Lgals*7 (71).

Members of the human galectin gene family are found in different chromosomes, including chromosome 1, 11, 14, 17, 19, and 22. Than et al. proposed that some of the human galectin genes clustered in chromosome 19 and expressed in villous trophoblasts, including *LGALS13, LGALS14, LGALS16*, are developmentally regulated by DNA methylation and induced by transcription factors that drive villous trophoblast differentiation and trophoblast-specific gene expression (49). In addition, dysregulation of these galectin genes with a potential role in immune tolerance to the semi-allogeneic fetus was proposed to be associated with preeclampsia (49).

Interestingly, the *Lgals*3 gene is different from other galectin genes in which gene duplication and inversion within a cluster has been reported. A single member has been identified per species hinting at a conserved function of Lgals3 during evolution (42). In addition, *LGALS3* contains an internal gene, which is much less abundant than *LGALS3* transcripts and is expressed mostly in peripheral blood leukocytes producing an entirely distinct protein from Lgals3 (72).

## Role of Galectins in Pregnancy Associated Processes

Expression profiling studies in reproductive tissues have shed important insights on the biological roles played by galectins in pregnancy orchestration, highlighting the importance of a delicate interplay between maternal and fetal sources of galectin expression for healthy outcomes (Figure 1). The following section provides a brief overview of the role of individual galectins expressed at the maternal-fetal interface in the establishment and maintenance of pregnancy.

## Galectin-1 (Lgals1)

The functions of Lgals1 in the context of pregnancy are the best characterized when compared to other members of the galectin family (Figure 1), likely due to its high level of expression by decidual stromal cells and trophoblast cell populations which suggested an important function (73). Indeed, Lgals1 has been shown to play a role in a variety of biological processes highly relevant for pregnancy orchestration including angiogenesis, immune response regulation, cell adhesion, invasion, and cell cycle progression through intracellular or extracellular mechanisms (23, 74–76).

Lgals1 expression is observed in 3–5 days human embryos potentially increasing trophoblast attachment to the uterine epithelium (77). After embryo attachment as the trophoblast layer differentiates, Lgals1 localizes to villous cytotrophoblast where it may play a role in promoting syncytium formation, although this function has only been studied *in vitro* using the BeWo trophoblast tumor cell line (78, 79). More recently, Lgals1 has been demonstrated to drive the differentiation of mouse trophoblast stem (TS) cells *in vitro*, by enhancing cell migration and invasiveness associated with a shift in the expression of matrix metalloproteinases, epithelial-mesenchymal transition markers and the TGF-β signaling pathway (80). Circulating levels of Lgals1 increase significantly during pregnancy and several studies indicate the potential use of Lgals1 as a biomarker for miscarriage, recurrent fetal loss and preeclampsia (PE) (77, 81–84). Whether circulating Lgals1 retains carbohydrate-binding activity within the oxidative nature of the extracellular environment remains unknown as Lgals1 exhibits exquisite sensitivity to oxidative inactivation (25, 85). In addition, a further question regarding concentration of galectins in serum or plasma is whether high picomolar concentrations are sufficient for galectins to act at a distance similar to circulating hormones (86). In this regard, some galectin-mediated cellular activities (e.g., Lgals3 and Lgals7) (87, 88) might be sufficiently sensitive to be elicited by serum levels of galectins.

*Lgals1* is highly expressed in the most invasive trophoblast cells of the placenta and membrane bound Lgals1 has been proposed to regulate migration of primary trophoblasts and of an extravillous trophoblast (EVT) cell line (77, 89–91). Modulation of EVT migration by Lgals1 could be related to its interaction with the β1 integrin chain on the EVT membrane (90, 92–94, 94). Another reported ligand for Lgals1 on the EVT membrane is the mucin MUC1 (95). Expression of *MUC1* is increased during placental development and was found to be elevated in severe pre-eclamptic placentas (96) although the significance of this finding is unclear as MUC1 has been shown to have adhesive and anti-adhesive properties (97). Interestingly, adhesion and invasion of the HTR-8 SV/neo EVT cell line to ECM components is negatively affected by *MUC1* overexpression (98). In endothelial cells, the membrane protein neuropilin-1 was identified as a ligand for Lgals1 and the expression of neuropilin-1 in decidual cells, intermediate trophoblasts, and syncytiotrophoblasts has been recently reported (99, 100). The potential interaction of Lgals1 with neuropilin-1 in these placental cells could potentially have functional consequences for placentation. As stated above, besides interacting with glycoproteins on the cell membrane, Lgals1 interacts with glycoproteins deposited in the ECM and has been shown to have both anti-adhesive as well as pro-adhesive extracellular functions (23). In the placental ECM, Lgals1 ligands include fibronectin, laminin, and osteopontin, which are also integrin ligands (19, 101–104).

The importance of Lgals1 as a contributor to feto-maternal tolerance has been described by many investigators and has been extensively reviewed (51). Several immune cells with essential roles in the establishment and maintenance of pregnancy synthesize and respond to Lgals1, e.g., CD4+ CD25+ regulatory T-cells, which play a very important role in tolerating the immunogenic paternal alloantigens (83, 105–110). In addition, *in vitro* studies showed that Lgals1 regulates the expression of human leucocyte antigen (HLA-G) in EVTs demonstrating that Lgals1 contributes to tolerance via its interaction with immune and trophoblast cells (77).

## Galectin-2 (Lgals2)

Lgals2 is predominantly expressed in the gastrointestinal tract and has been identified as one of the main gastric mucosal proteins proposed to have a protective role in the stomach by interacting with mucins (111). In addition, immune functions of Lgals2 have been proposed including its ability to induce apoptosis in activated CD8^+^ T-cells and its effects on monocytes (112, 113). Lgals2 was shown to polarize monocytes and macrophages to a pro-inflammatory, non-arteriogenic M1 phenotype, and reduce monocyte motility. Interestingly, Lgals2 regulation of monocyte/macrophage phenotype were attributed to its interaction with the lipopolysaccharide-binding protein CD14 in a non-carbohydrate dependent manner (112, 113). Lgals2 expression in the placenta was reported in both VT and EVT cells and was shown to be expressed at higher levels in VT and EVT of male compared to female placentas. Interestingly, in cases of intrauterine growth restriction (IUGR), there was no change in expression in female placentas compared to controls; however, expression of Lgals2 in male IUGR placentas was reported to be decreased compared to controls (114). While these studies should be repeated with a larger sample number, they may serve to caution investigators for the need to take the gender of the fetus into account as an important variable when analyzing possible changes in galectin expression when comparing normal to pathologic pregnancies. In addition, *Lgals2* expression was decreased in third-trimester EVT trophoblast cells in cases of PE on the protein and mRNA level (115) and also significantly down-regulated in the VT and EVT trophoblast of spontaneous and recurrent abortion placentas (116).

## Galectin-3 (Lgals3)

Lgals3 has been implicated in the regulation of innate and adaptive immune responses, where it participates in the activation or differentiation of immune cells and contributes to phagocytic clearance of microorganisms and apoptotic cells by macrophages (117, 118). Lgals3 has been reported to promote but also to inhibit T-cell apoptosis depending on whether it binds to glycoproteins on the cell surface (CD45 and CD71) or to intracellular ligands (Bcl-2) (119, 120). In the placenta, Lgals3 was detected in all trophoblastic lineages including villous cytotrophoblasts (CTB) and EVT with a reduction of Lgals3 expression observed from the VT to the trophoblastic cell columns (121). This pattern of Lgals3 expression correlates with the switch from a proliferative to a migratory trophoblast phenotype and while placental Lgals3 dysregulation has been associated with some obstetric complications including spontaneous or recurrent miscarriages, further studies are needed to understand its contribution to trophoblast biology (81, 122). In addition to trophoblasts, Lgals3 is expressed by maternal decidual cells (73). While showing a different expression pattern, both Lgals1 and Lgals3 have been proposed to play a role in cell-cell and cell-matrix interactions of trophoblast during placentation (121). Studies of the importance of Lgals3 in murine pregnancy by Yang et al. indicate that Lgals3 is expressed in the luminal and glandular epithelium and that an increase in Lgals3 is required for proper embryo implantation (123). In addition, Lgals3 affects chemotaxis and morphology of endothelial cells and stimulates capillary tube formation and angiogenesis *in vivo* (124). Therefore, besides its proposed roles in embryo implantation, immune regulation and trophoblast-matrix interactions, Lgals3 has a potential role in placental angiogenesis. It must be noted, however, that despite considerable research efforts over the past years, the precise physiological relevance of this lectin during pregnancy remains ill-defined. Comprehensive analysis of the placental phenotype, the regulation of vascular development and maternal adaptations in *Lgals3* deficient models could greatly aid our understanding of this lectin's role in pregnancy orchestration.

## Galectin-7 (Lgals7)

Lgals7 is produced by the premenstrual and menstrual endometrial luminal and glandular epithelium, where it accumulates in menstrual fluid and has been proposed to act as a paracrine factor to facilitate post-menstrual endometrial re-epithelialization (125). While *Lgals7* mRNA was not detected in term placenta by real time-PCR, using immunohistochemistry, expression of Lgals7 was reported in the syncytiotrophoblast (STB), EVT and glandular epithelium in first trimester placenta, decidua and in the STB and endothelial cells of normal term placenta (50, 126). Menkhorst et al. suggested that Lgals7 may facilitate adhesion of the embryo to the endometrium and reported that the serum concentration of Lgals7 was significantly elevated in women (weeks 10–12 and 17–20) who subsequently developed PE compared to women with healthy pregnancies (126, 127). Another study, also explored the potential value of Lgals7 measurement as a biomarker and indicated that maternal serum Lgals7 levels had no value to predict the risk of spontaneous abortion (128). Clearly, further studies are required to confirm the expression of Lgals7 in placental cells and the potential usefulness of Lgals7 measurements in maternal serum as a biomarker for pregnancy pathologies should be evaluated with larger patient cohorts.

## Galectin-8 (Lgals8)

*Lgals8* is ubiquitously expressed and analysis of its expression in normal first trimester placentas indicated that *Lgals8* is expressed by VT and EVT, and is highly expressed in decidual stromal cells (129). Lgals8 has been referred to as an “angiogenesis regulator” in vascular and lymphatic endothelium by binding to podoplanin in lymphatic vessels and CD166 (ALCAM, activated leukocyte cell adhesion molecule) in vascular endothelial cells (130). The role of this galectin in placental angiogenesis has not been explored but an initial report indicates that Lgals8 is not expressed in the endothelium of the placenta (130). The human gene (*LGALS8*) encodes seven different isoforms resulting from alternative splicing but the functional consequences of *Lgals8* splicing are poorly understood (130). Pro-inflammatory and immunosuppressive functions have been both attributed to this galectin in different experimental systems but so far, no studies on its possible role as an immunomodulator during pregnancy have been reported (131–135). Potentially, Lgals8 could play a role in trophoblast cell adhesion and migration as was reported in other cell types, but experimental evidence for the regulation of trophoblast function by this lectin is also lacking (136).

## Galectin-9 (Lgals9)

The tandem-repeat Lgals9 has been implicated in immune regulation through binding to TIM-3, CD44 and the cell surface protein disulfide isomerase (PDI) (137–139). This galectin is expressed by many cell types including epithelial cells of the endometrium, trophoblasts, stromal cells of the decidua, endothelial cells including those in the placenta, and several types of immune cells (140–143). Splice variants of *Lgals9* have been reported with six of them expressed in human decidua, which may differ in their biological functions (143–145).

Compared to non-pregnant individuals, regulatory T cells show higher level of *Lgals9* expression as pregnancy proceeds and the level of Lgals9 in serum is significantly higher in women with normal pregnancies compared to post-partum and non-pregnant female controls (146). Interestingly, the serum levels of Lgals9 in pregnancy varied with the gender of the fetus as was also reported for some inflammatory cytokines and pro-angiogenic factors; Lgals9 is further increased in the serum of women carrying a male compared to a female fetus (147, 148). Li et al. proposed that Lgals9 contributes to the generation of CD25^+^ FoxP3^+^ T regulatory cells in -circulation and in the spleen and that engagement of Tim-3 by Lgals9 in peripheral NK cells facilitates the immunosuppressive activity of these cells during the first trimester of pregnancy (149). Additionally, they report that the concentration of Lgals9 in the plasma of women with normal pregnancies is significantly higher from that in women suffering from recurrent miscarriages but caution should be taken as the sample size was small and fetal gender was not considered in these studies (149).

Recently, lower levels of Lgals9 expression analyzed by immunohistochemistry were described in trophoblasts of the DBA/2-mated CBA/J mouse model of spontaneous abortion/PE when compared to normal CBA/J × BALB/c matings, further showing that Lgals9 blockade promoted a significant imbalance of Th1/Th2 immunity in this model (150). Additionally, altered placental Lgals9 expression together with dysregulated Tim-3 signaling in distinct NK and T cell subsets have been suggested to mediate the abortifacient effects of mifepristone in mouse pregnancies (151). Furthermore, activation of Tim-3/Lgals9 signaling pathway promotes decidual macrophages polarization to M2 subtype, alleviating the PE-like syndrome induced by LPS in a rat model (152). In conclusion, while a role for Lgals9 in immune tolerance during pregnancy has been reported by a handful of investigators, more studies considering splice variants, fetal gender, and Lgals9 receptors on target cells are required to better understand the potential role for this galectin as a contributor of the systemic and local immune regulation during pregnancy.

## Galectin-10 (Lgals10)

Prototype Lgals10, also known as eosinophil Charcot-Leyden crystal protein, appears to play an important role in the differentiation of neutrophils and the functional properties of CD25+Treg cells (153, 154). Subsequently, expression of this lectin at the maternal-fetal interface has been described mainly in the STB and to a smaller extent in the decidua during the first trimester, showing decreased levels in spontaneous abortion patients (116, 155). The precise physiological role played by this lectin in pregnancy is still unknown but interestingly, its expression is driven from a chromosome 19 gene cluster comprising also galectins −13, −14, −16, and −17, which emerged during primate evolution as a result of duplication and rearrangement of genes via a birth-and-death process (49, 50). Galectins in the chromosome 19 cluster show primarily placental expression and may be involved in the regulation of unique pregnancy associated processes, including maternal immune tolerance and villous trophoblast differentiation (49, 50, 156).

## Galectin 13 (Lgals13)

Lgals13 is also known as placental protein 13 (PP13) and was first isolated from human placenta (157). This galectin is predominantly expressed by STB cells of the placenta, in which nuclear staining and strong labeling of the brush border membrane is observed (158, 159). Although originally reported to be absent in serum of pregnant women, Lgals13 is detected in increasing concentration in maternal serum as pregnancy progresses becoming undetectable 2–5 weeks post-partum (158, 160, 161). Besides being found in a soluble form in circulation, Lgals13 is also located inside and on all types of STB-derived extracellular vesicles (162).

Lgals13 has been proposed to have immune regulatory functions, and in studies in rodents it has been shown to reduce blood pressure associated with activation of endothelial prostaglandin and nitric oxide signaling pathways (163–166). The potential for Lgals13 as a useful biomarker for PE has been suggested by Burger and co-workers. They reported that in the 1st trimester, lower than normal Lgals13 levels were found in IUGR and PE, particularly in the early-onset form. In the 2nd and 3rd trimesters, higher than normal concentrations were found in PE, IUGR and in preterm delivery (PTD) (160). On the other hand, lower placental *Lgals13* mRNA and protein expression were found in preterm PE and HELLP syndrome, although the immunoreactivity of the STB microvillous membrane was reported to be stronger in these pregnancies than in age-matched controls suggesting increased membrane shedding (167). The usefulness of Lgals13 as a biomarker for PE has been questioned as late second-trimester Lgals13 alone does not increase the ability to predict PE when compared to second-trimester Doppler pulsatility index and other potential biochemical markers (168), highlighting the need to consider the interactions between different signaling pathways in disease pathogenesis when in search for sensitive, reliable biomarkers. In this regard, recent studies suggest that the kinetics of Lgals13 expression in PE would result from the concerted actions of this protein and antiangiogenic factors as sFlt-1 on the maternal vascular system, with a dual role for Lgals13: first in low levels acting as a priming insult promoting endothelial activation and angiogenic imbalance, and increasing later in the third trimester as a natural rescue response promoting maternal vasodilation to lower blood pressure (169). In the context of gestational diabetes mellitus (GDM), increased Lgals13 serum levels during the early second trimester and lower expression in trophoblast cells of the term placenta have been reported (170, 171). Dysregulation of *Lgals13* was suggested to contribute to an imbalance in inflammatory processes in the placenta during pregnancy and therefore possibly lead to GDM.

Interestingly, while Lgals13 has hemagglutination activity when tested with chicken erythrocytes, a recent report suggested that contrary to what was observed for other galectins, Lgals13 may not bind carbohydrates (172). Prior studies, however, had reported that not only the binding of Lgals13 to erythrocytes or T cells is carbohydrate-dependent (50, 173), but also that N-acetyllactosamine is the preferred disaccharide ligand for Lgals13 (50, 159). This inconsistency in the results from the aforementioned laboratories may reside in that Su et al. (172) had tested lactose as an inhibitor for Lgals13, as well as other carbohydrates, such as xylose and arabinose, that are unrelated to the structures recognized by most galectins. Nevertheless, the identification of natural ligand(s) for Lgals13 at the cell surface and extracellular matrix will be of great importance to better understand its role(s) during pregnancy (165).

## Galectin-Glycan Interactions as Regulators of the Fetal-Maternal Dialogue

**Figure 2:**
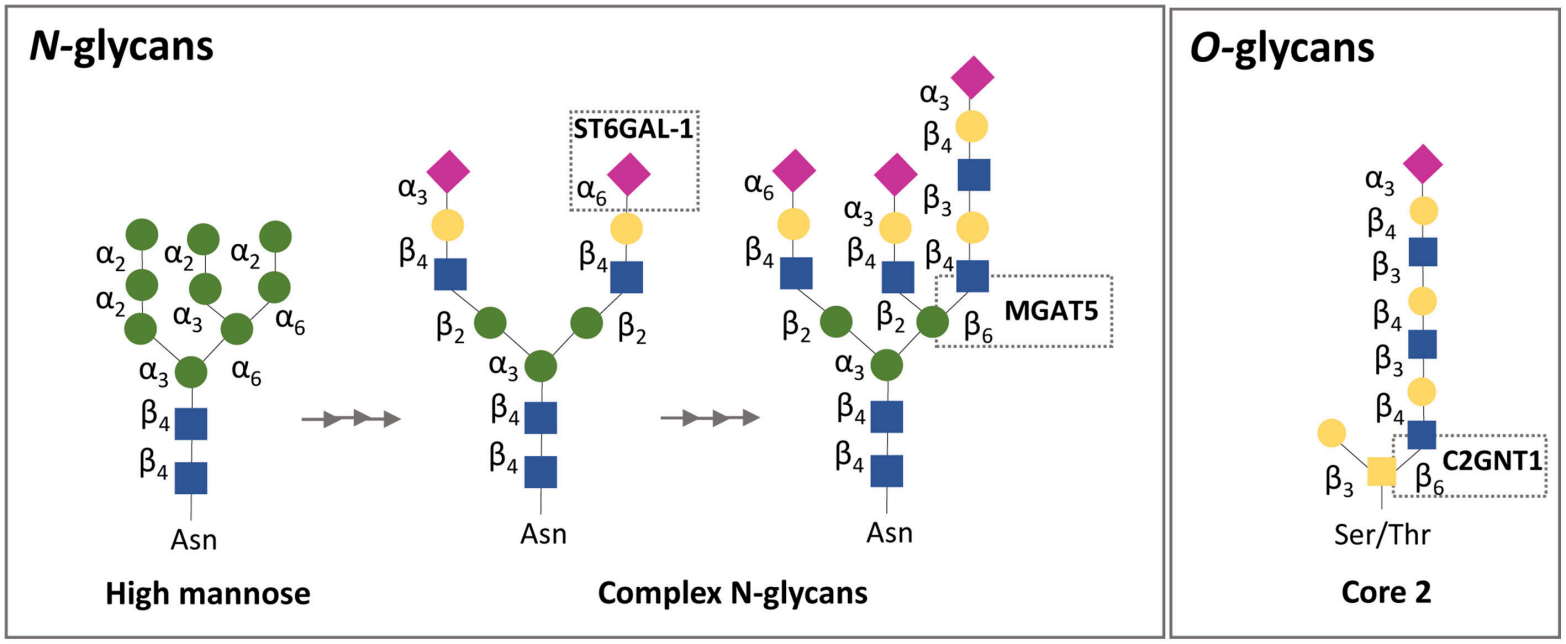
Simplified schematic representation of *N*-and *O*-glycan biosynthesis focusing on galectin-1 binding affinity. N-glycans are attached to asparagine (Asn) residues, whereas O-glycans are attached to either serine (Ser) or threonine (Thr) residues. Gal-1 recognizes galactose on complex *N*-glycans and sialylation on the terminal galactose in the α2,6-linkage, but not in the α2,3-linkage, prevents the binding of gal-l. Regarding *O*-glycans, gal-1 binds to the N-acetyllactosamine (LacNAc) motif in core 2 *O*-glycans. ST6GAL-1, β-galactoside α2,6-sialyltransferase 1; *MGATS*, α1,6-mannosylglycoprotein 6β-N-acetylglucosaminyltransferase; *C2GnT*, core 2 β1,6 N-acetylglucosaminyltransferase.

Glycosylation is the most common and structurally diverse type of post-translational modification, affecting proteins, lipids and the extracellular matrix. Glycans play fundamental roles in most biological processes, thus it is not surprising that glycans are profusely expressed in the mammalian uterus (174). During implantation, the uterine epithelium and the outer trophoblast cell layer of the implanting embryo interact in a glyco-specific manner, such that perturbations of the system generally result in failure of implantation or poor placentation and compromised pregnancy outcomes. Glycans are essential functional groups that facilitate and influence the reproduction process. The synthesis of glycans relies on specific modification enzymes (glycosyltransferases and glycosidases) (Figure 2), and the glycocode expressed in a particular tissue is highly dependent on the cell type and its developmental, nutritional, and pathological state. The glycans within the glycome can have multiple functions during pregnancy. For example, N-linked glycans (attached to the nitrogen of an asparagine side-chain) play an important role in trophoblast cell invasion in early pregnancy (175, 176) and maternal-fetal tolerance (177, 178). O-Linked glycans (attached to the hydroxyl oxygen of serine, threonine, tyrosine, hydroxylysine or hydroxyproline side-chains) can influence recognition events during fertilization (e.g., sperm-egg interactions) (179). As a detailed discussion about the role of glycosylation—in pregnancy outcome is beyond the scope of this review, we will focus on modifications that affect binding and function of members of the galectin family during gestation.

**Figure 3:**
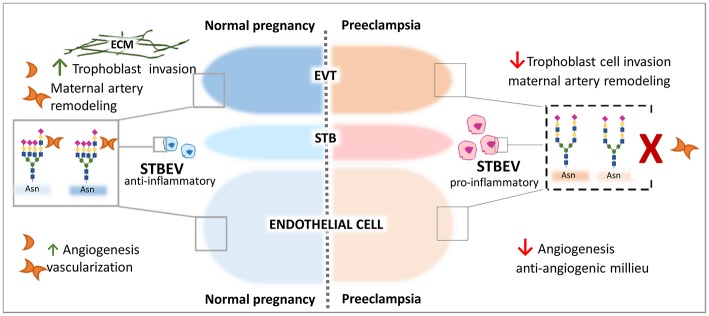
Galectin-1-Giycan axis in the control of pregnancy protective programs. Galectin-1 regulates key process during the course of normal gestation including trophoblast invasion, maternal immune regulation, and angiogenesis. Relevant examples are illustrated. During Preeclampsia an aberrant α2-6 sialylation decorates α5β1integrin on the cell surface of EVT trophoblast, cell surface of STVEV released from the STB trophoblast and on endothelial cells. The high expression of α2-6 sialylated *N*-glycans impairs gal-1-mediated trophoblast ETV cell migration process interfering with the binding to the ECM and subsequently invasion. High α2-6 sialylation on STBEV and impaired gal-l binding might contribute to the pro-inflammatory milieu in maternal circulation and endothelial dysfunction. On vascular endothelial cells, the aberrant α2-6 sialylation may disrupt gal-1-mediated angiogenesis and early vascularization promoting the anti-angiogenesis status typical of the syndrome.

Extracellularly galectins act by cross-linking *N-* and *O-*glycans on the surface glycoproteins of maternal immune cells, trophoblasts and endothelial cells at the fetal-maternal interface. As glycosylation often represent highly regulated post-translational modifications related to the physiological cellular status, alterations in glycan composition can fundamentally impact galectin activity (180–182). Given the prominent expression of galectins (e.g., Lgals1 and Lgals3) at the fetal-maternal interface, studies designed to examine the relevance of cell surface glycans on maternal/ placental compartments are of key importance. In a physiological context, enhanced expression of N-acetylglucosaminyl transferase V [GnTV, encoded by the *Mgat5* gene (Figure 2)] was found in placentas from the first trimester compared with those from full-term pregnancies (175). GnTV generates β1-6-N-acetylglucosamine branches in complex N-glycans which are recognized by Lgals1. LacNAc motives are a glycan signature of EVT (91, 183) as their presence was detected not only on their surface but also on their secretion product HLA-G (91, 184). Since Lgals1 promotes trophoblast invasion and EVT differentiation during early pregnancy (90), it is possible that the increased activity of GnTV results in enhanced Lgals1 signaling (176); particularly, by promoting the interaction of Lgals1 with β1 integrin at the trophoblast cell membrane (94, 176, 185–190) (Figure 3). Furthermore, the presence of (β-6) branches and the expression of the glycosyltransferase GnTV involved in the generation of these glycan structures were reduced in villous tissues from early spontaneous miscarriages in comparison with healthy pregnancy villous tissues (191). Thus, differences in the glycan composition of trophoblast related-proteins at the same gestational age could be important disease biomarkers that should be further explored with newly available mass spectroscopy techniques. Indeed, the expression of GnTV was reported to be elevated in PE placentas compared to normal placentas (192). We have shown that *Lgals1* expression is increased in late onset PE and could represent a compensatory mechanism of the trophoblast to overcome the severe inflammation microenvironment that characterizes PE disease (74) (Figure 3). This is an interesting link to the metabolic status of trophoblast cells, which is mediated by the intracellular levels of GnTV that affect quality and branching of complex N-glycans and therefore regulate galectin binding.

During pregnancy the STB layer of the placenta releases extracellular vesicles (STBEV) containing a complex cargo of RNAs, proteins, lipids, and also glycans into the maternal circulation potentially to induce maternal immune adaption. Under pathological conditions such as preeclampsia, however, STBEV exhibit a differential glycan composition compared to uneventful pregnancies. In particular, STBEV derived from PE placentas depict an increased content α2-6-linked sialic acid (193). The presence of α2-6-linked sialic acid on cell surface glycoproteins—is mainly determined—by the activity of the sialyltransferase gene *ST6GAL1* and results in blocking of Lgals1 signaling (194, 195). The selective glycosyltransferase expression (e.g., ST6Gal-1) on trophoblast cells may be an early pathological mechanism of masking Lgals1 activity in modulating the maternal immune response to the developing embryo. Moreover, an increased α2-6 sialylation was observed on the STB layer and also in placenta vessels derived from pregnancies complicated with hypertensive disorders including superimposed PE, PE, and PE + HELLP (196). This is important since high α2-6 sialylation on endothelial cells can reduce Lgals1 mediated angiogenesis (195), which is in line with our *in vivo* experiment showing that blocking Lgals1 mediated angiogenesis with anginex during early gestation in mice induced spontaneously PE development (74) (Figure 3). Moreover, the inhibition of Lgals1 binding by sialylation at the position 6 of galactose has been suggested to make Th1 cells resistant to apoptosis (197) and might contribute to uncontrolled maternal inflammation during preeclampsia. Thus, analysis of the glycosylation signature of trophoblast and placental vessels constitute a valuable approach to unravel the importance of galectin signaling through VEGFR2 during gestation.

## Conclusions and Future Directions

There is ample evidence showing that galectins are expressed widely at the feto-maternal interface. Their expression is regulated during pregnancy and galectins are highly specific to certain trophoblast and maternal cell types. Multiple galectin functions have been described in the orchestration of healthy pregnancy, which include maternal immune adaptation, placental development, and angiogenesis. Studies on the association of pregnancy pathologies with dysregulated galectin expression are still at an early stage, with most of our knowledge on the biological role of galectins in pregnancy being inferred from *in vitro* models and clinical correlations. However, sufficient evidence is already available to suggest galectins, especially Lgals1 and Lgals13, are promising candidates for further investigation aimed at understanding the pathogenesis of pregnancy complications including life threatening pregnancy related diseases such as PE. Because galectins are unique proteins with ability to recognize and decode a complex array of glycan motifs, future research could include: (1) a systemic study of the trophoblast cell-type glycome and galectin expression at the maternal-fetal interface in health and disease to determine whether glycomodifications on trophoblast cells that prevent galectin binding are responsible for the development of some pregnancy disorders and what is the galectin distribution in the maternal and placental compartments in health and disease; (2) a comprehensive analysis of the role of galectins in maternal circulation during pregnancy to establish whether galectins act at distance and if the presence of galectins in maternal circulation is a consequence of leakage from placenta tissue; (3) a deep analysis of galectin-glycan interactions either at the maternal or placental compartments with the goal to reveal the critical contribution of the physiological and pathophysiological galectin functions during gestation. In the years ahead, the development of novel *in vivo* strategies to test hypotheses related to the biology of galectin-glycan interactions during pregnancy represents a worthwhile pursuit, which will greatly advance reproductive medicine

## Author Contributions

All authors listed have made a substantial, direct and intellectual contribution to the work, and approved it for publication.

### Conflict of Interest Statement

The authors declare that the research was conducted in the absence of any commercial or financial relationships that could be construed as a potential conflict of interest.

## References

[1] Hyde KJ Schust DJ. Immunologic challenges of human reproduction: an evolving story. Fertil Steril. (2016) 106:499–510. 10.1016/j.fertnstert.2016.07.1073 27477190

[2] Cross JC Hemberger M Lu Y Nozaki T Whiteley K Masutani M. Trophoblast functions, angiogenesis and remodeling of the maternal vasculature in the placenta. Mol Cell Endocrinol. (2002) 187:207–12. 10.1016/S0303-7207(01)00703-1 11988329

[3] Norwitz ER. Defective implantation and placentation: laying the blueprint for pregnancy complications. Reprod Biomed Online. (2007) 14:101–9. 10.1016/S1472-6483(10)61464-2 20483405

[4] Barker DJ. *In utero* programming of chronic disease. Clin Sci. (1998) 95:115–28. 10.1042/cs0950115 9680492

[5] Jansson T Powell TL. Role of the placenta in fetal programming: underlying mechanisms and potential interventional approaches. Clin Sci. (2007) 113:1–13. 10.1042/CS20060339 17536998

[6] Cheong JN Wlodek ME Moritz KM Cuffe JS. Programming of maternal and offspring disease: impact of growth restriction, fetal sex and transmission across generations. J Physiol. (2016) 594:4727–40. 10.1113/JP271745 26970222 PMC5009791

[7] Fajersztajn L Veras MM. Hypoxia: from placental development to fetal programming. Birth Defects Res. (2017) 109:1377–85. 10.1002/bdr2.1142 29105382

[8] Kasai K Hirabayashi J. Galectins: a family of animal lectins that decipher glycocodes. J Biochem. (1996) 119:1–8. 10.1093/oxfordjournals.jbchem.a021192 8907168

[9] Andre S Kaltner H Manning JC Murphy PV Gabius HJ. Lectins: getting familiar with translators of the sugar code. Molecules. (2015) 20:1788–823. 10.3390/molecules20021788 25621423 PMC6272290

[10] Vasta GR Ahmed H Animal Lectins: A Functional View. (2008). Boca Raton, FL: CRC Press.

[11] Zelensky AN Gready JE. The C-type lectin-like domain superfamily. FEBS J. (2005) 272:6179–217. 10.1111/j.1742-4658.2005.05031.x 16336259

[12] Vasta GR Amzel LM Bianchet MA Cammarata M Feng C Saito K. F-type lectins: a highly diversified family of fucose-binding proteins with a unique sequence motif and structural fold, involved in self/non-self-recognition. Front Immunol. (2017) 8:1648. 10.3389/fimmu.2017.01648 29238345 PMC5712786

[13] Cooper DN. Galectinomics: finding themes in complexity. Biochim Biophys Acta. (2002) 1572:209–31. 10.1016/S0304-4165(02)00310-0 12223271

[14] Cummings RD Liu FT Vasta GR Chapter 36: Galectins. In: Varki A Cummings RD Esko JD Stanley P Hart GW Aebi M Darvill AG Kinoshita T Packer NH Prestegard JH Schnaar RL Seeberger PH, editors. Essentials of Glycobiology, 3rd ed. Cold Spring Harbor, NY: Cold Spring Harbor Laboratory Press (2015–2017) p. 469–80.

[15] Hirabayashi J Hashidate T Arata Y Nishi N Nakamura T Hirashima M. Oligosaccharide specificity of galectins: a search by frontal affinity chromatography. Biochim Biophys Acta. (2002) 1572:232–54. 10.1016/S0304-4165(02)00311-2 12223272

[16] Cho M Cummings RD Galectin-1, a beta-galactoside-binding lectin in Chinese hamster ovary cells. II Localization and biosynthesis. J Biol Chem. (1995) 270:5207–12. 10.1074/jbc.270.10.5207 7890631

[17] Tsay YG Lin NY Voss PG Patterson RJ Wang JL. Export of galectin-3 from nuclei of digitonin-permeabilized mouse 3T3 fibroblasts. Exp Cell Res. (1999) 252:250–61. 10.1006/excr.1999.4643 10527616

[18] Cooper DN Barondes SH. Evidence for export of a muscle lectin from cytosol to extracellular matrix and for a novel secretory mechanism. J Cell Biol. (1990) 110:1681–91. 10.1083/jcb.110.5.1681 2335567 PMC2200163

[19] Ozeki Y Matsui T Yamamoto Y Funahashi M Hamako J Titani K. Tissue fibronectin is an endogenous ligand for galectin-1. Glycobiology. (1995) 5:255–61. 10.1093/glycob/5.2.255 7780201

[20] Parsons MJ Pollard SM Saude L Feldman B Coutinho P Hirst EM. Zebrafish mutants identify an essential role for laminins in notochord formation. Development. (2002) 129:3137–46. 12070089 10.1242/dev.129.13.3137

[21] Nabi IR Shankar J Dennis JW. The galectin lattice at a glance. J Cell Sci. (2015) 128:2213–9. 10.1242/jcs.151159 26092931

[22] Piyush T Chacko AR Sindrewicz P Hilkens J Rhodes JM Yu LG. Interaction of galectin-3 with MUC1 on cell surface promotes EGFR dimerization and activation in human epithelial cancer cells. Cell Death Differ. (2017) 24:1937–47. 10.1038/cdd.2017.119 28731466 PMC5635220

[23] Elola MT Wolfenstein-Todel C Troncoso MF Vasta GR Rabinovich GA. Galectins: matricellular glycan-binding proteins linking cell adhesion, migration, and survival. Cell Mol Life Sci. (2007) 64:1679–700. 10.1007/s00018-007-7044-8 17497244 PMC11136145

[24] Markowska AI Cao Z Panjwani N. Glycobiology of ocular angiogenesis. Glycobiology. (2014) 24:1275–82. 10.1093/glycob/cwu078 25108228 PMC4211602

[25] Stowell SR Cho M Feasley CL Arthur CM Song X Colucci JK. Ligand reduces galectin-1 sensitivity to oxidative inactivation by enhancing dimer formation. J Biol Chem. (2009) 284:4989–99. 10.1074/jbc.M808925200 19103599 PMC2643495

[26] Lobsanov YD Gitt MA Leffler H Barondes SH Rini JM. X-ray crystal structure of the human dimeric S-Lac lectin, L-14-II, in complex with lactose at 2.9-A resolution. J Biol Chem. (1993) 268:27034–8. 10.2210/pdb1hlc/pdb 8262940

[27] Liao DI Kapadia G Ahmed H Vasta GR Herzberg O. Structure of S-lectin, a developmentally regulated vertebrate beta-galactoside-binding protein. Proc Natl Acad Sci USA. (1994) 91:1428–32. 10.1073/pnas.91.4.1428 8108426 PMC43172

[28] Di Lella S Marti MA Croci DO Guardia CM Diaz-Ricci JC Rabinovich GA. Linking the structure and thermal stability of beta-galactoside-binding protein galectin-1 to ligand binding and dimerization equilibria. Biochemistry. (2010) 49:7652–8. 10.1021/bi100356g 20666428

[29] Lopez-Lucendo MF Solis D Andre S Hirabayashi J Kasai K Kaltner H Growth-regulatory human galectin-1: crystallographic characterization of the structural changes induced by single-site mutations and their impact on the thermodynamics of ligand binding. J Mol Biol. (2004) 343:957–70. 10.1016/j.jmb.2004.08.078 15476813

[30] Vasta GR. Roles of galectins in infection. Nat Rev Microbiol. (2009) 7:424–38. 10.1038/nrmicro2146 19444247 PMC3759161

[31] Cerliani JP Stowell SR Mascanfroni ID Arthur CM Cummings RD Rabinovich GA. Expanding the universe of cytokines and pattern recognition receptors: galectins and glycans in innate immunity. J Clin Immunol. (2011) 31:10–21. 10.1007/s10875-010-9494-2 21184154

[32] Vasta GR. Galectins as pattern recognition receptors: structure, function, and evolution. Adv Exp Med Biol. (2012) 946:21–36. 10.1007/978-1-4614-0106-3_2 21948360 PMC3429938

[33] Thiemann S Baum LG. Galectins and immune responses-just how do they do those things they do? Annu Rev Immunol. (2016) 34:243–64. 10.1146/annurev-immunol-041015-055402 26907217

[34] Sato S St-Pierre C Bhaumik P Nieminen J. Galectins in innate immunity: dual functions of host soluble beta-galactoside-binding lectins as damage-associated molecular patterns (DAMPs) and as receptors for pathogen-associated molecular patterns (PAMPs). Immunol Rev. (2009) 230:172–87. 10.1111/j.1600-065X.2009.00790.x 19594636

[35] Walser PJ Kues U Aebi M Kunzler M. Ligand interactions of the *Coprinopsis cinerea* galectins. Fungal Genet Biol. (2005) 42:293–305. 10.1016/j.fgb.2004.12.004 15749049

[36] Stalz H Roth U Schleuder D Macht M Haebel S Strupat K. The *Geodia cydonium* galectin exhibits prototype and chimera-type characteristics and a unique sequence polymorphism within its carbohydrate recognition domain. Glycobiology. (2006) 16:402–14. 10.1093/glycob/cwj086 16449348

[37] Saouros S Edwards-Jones B Reiss M Sawmynaden K Cota E Simpson P. A novel galectin-like domain from *Toxoplasma gondii* micronemal protein 1 assists the folding, assembly, and transport of a cell adhesion complex. J Biol Chem. (2005) 280:38583–91. 10.1074/jbc.C500365200 16166092

[38] Huynh J Dawson D Roberts D Bentley-Lewis R. A systematic review of placental pathology in maternal diabetes mellitus. Placenta. (2015) 36:101–14. 10.1016/j.placenta.2014.11.021 25524060 PMC4339292

[39] Dormitzer PR Sun ZY Wagner G Harrison SC. The rhesus rotavirus VP4 sialic acid binding domain has a galectin fold with a novel carbohydrate binding site. EMBO J. (2002) 21:885–97. 10.1093/emboj/21.5.885 11867517 PMC125907

[40] Monnier N Higo-Moriguchi K Sun ZY Prasad BV Taniguchi K Dormitzer PR. High-resolution molecular and antigen structure of the VP8^*^ core of a sialic acid-independent human rotavirus strain. J Virol. (2006) 80:1513–23. 10.1128/JVI.80.3.1513-1523.2006 16415027 PMC1346936

[41] Sun X Li D Peng R Guo N Jin M Zhou Y Functional and structural characterization of P[19] rotavirus VP8^*^ interaction with histo-blood group antigens. J Virol. (2016) 90:9758–65. 10.1128/JVI.01566-16 27535055 PMC5068527

[42] Houzelstein D Goncalves IR Fadden AJ Sidhu SS Cooper DN Drickamer K. (2004). Phylogenetic analysis of the vertebrate galectin family. Mol Biol Evol. 21:1177–87. 10.1093/molbev/msh082 14963092

[43] Hirabayashi J Kasai K. The family of metazoan metal-independent beta-galactoside-binding lectins: structure, function and molecular evolution. Glycobiology. (1993) 3:297–304. 10.1093/glycob/3.4.297 8400545

[44] Rabinovich GA Toscano MA Jackson SS Vasta GR. Functions of cell surface galectin-glycoprotein lattices. Curr Opin Struct Biol. (2007) 17:513–20. 10.1016/j.sbi.2007.09.002 17950594 PMC2100406

[45] Dam TK Brewer CF. Effects of clustered epitopes in multivalent ligand-receptor interactions. Biochemistry. (2008) 47:8470–6. 10.1021/bi801208b 18652478

[46] Hsu DK Chen HY Liu FT. Galectin-3 regulates T-cell functions. Immunol Rev. (2009) 230:114–27. 10.1111/j.1600-065X.2009.00798.x 19594632

[47] Hirashima M Kashio Y Nishi N Yamauchi A Imaizumi TA Kageshita T. Galectin-9 in physiological and pathological conditions. Glycoconj J. (2002) 19:593–600. 10.1023/B:GLYC.0000014090.63206.2f 14758084

[48] Heusschen R Schulkens IA Van Beijnum J Griffioen AW Thijssen VL. Endothelial LGALS9 splice variant expression in endothelial cell biology and angiogenesis. Biochim Biophys Acta. (2014) 1842:284–92. 10.1016/j.bbadis.2013.12.003 24333696

[49] Than NG Romero R Xu Y Erez O Xu Z Bhatti G. Evolutionary origins of the placental expression of chromosome 19 cluster galectins and their complex dysregulation in preeclampsia. Placenta. (2014b) 35:855–65. 10.1016/j.placenta.2014.07.015 25266889 PMC4203431

[50] Than NG Romero R Goodman M Weckle A Xing J Dong Z. A primate subfamily of galectins expressed at the maternal-fetal interface that promote immune cell death. Proc Natl Acad Sci USA. (2009) 106:9731–6. 10.1073/pnas.0903568106 19497882 PMC2689813

[51] Barrientos G Freitag N Tirado-Gonzalez I Unverdorben L Jeschke U Thijssen VL. Involvement of galectin-1 in reproduction: past, present and future. Hum Reprod Update. (2014) 20:175–93. 10.1093/humupd/dmt040 24077937

[52] Blois SM Barrientos G Galectin signature in normal pregnancy and preeclampsia. J Reprod Immunol. (2014) 101–2:127–34. 10.1016/j.jri.2013.05.005 23953090

[53] Craig SE Thummel R Ahmed H Vasta GR Hyde DR Hitchcock PF. The zebrafish galectin Drgal1-l2 is expressed by proliferating Muller glia and photoreceptor progenitors and regulates the regeneration of rod photoreceptors. Invest Ophthalmol Vis Sci. (2010) 51:3244–52. 10.1167/iovs.09-4879 20071673 PMC2891476

[54] Schwarz FP Ahmed H Bianchet MA Amzel LM Vasta GR. Thermodynamics of bovine spleen galectin-1 binding to disaccharides: correlation with structure and its effect on oligomerization at the denaturation temperature. Biochemistry. (1998) 37:5867–77. 10.1021/bi9716478 9558320

[55] Seetharaman J Kanigsberg A Slaaby R Leffler H Barondes SH Rini JM. X-ray crystal structure of the human galectin-3 carbohydrate recognition domain at 2.1-A resolution. J Biol Chem. (1998) 273:13047–52. 10.1074/jbc.273.21.13047 9582341

[56] Ideo H Seko A Ishizuka I Yamashita K. The N-terminal carbohydrate recognition domain of galectin-8 recognizes specific glycosphingolipids with high affinity. Glycobiology. (2003) 13:713–23. 10.1093/glycob/cwg094 12851289

[57] Ideo H Matsuzaka T Nonaka T Seko A Yamashita K. Galectin-8-N-domain recognition mechanism for sialylated and sulfated glycans. J Biol Chem. (2011) 286:11346–55. 10.1074/jbc.M110.195925 21288902 PMC3064191

[58] Krejcirikova V Pachl P Fabry M Maly P Rezacova P Brynda J. Structure of the mouse galectin-4 N-terminal carbohydrate-recognition domain reveals the mechanism of oligosaccharide recognition. Acta Crystallogr D Biol Crystallogr. (2011) 67:204–11. 10.1107/S0907444911004082 21358051

[59] Yoshino TP Dinguirard N Kunert J Hokke CH. Molecular and functional characterization of a tandem-repeat galectin from the freshwater snail *Biomphalaria glabrata*, intermediate host of the human blood fluke *Schistosoma mansoni*. Gene. (2008) 411:46–58. 10.1016/j.gene.2008.01.003 18280060 PMC2423817

[60] Shi XZ Wang L Xu S Zhang XW Zhao XF Vasta GR. A galectin from the kuruma shrimp (*Marsupenaeus japonicus*) functions as an opsonin and promotes bacterial clearance from hemolymph. PLoS ONE. (2014) 9:e91794. 10.1371/journal.pone.0091794 24618590 PMC3950279

[61] Vasta GR Feng C Bianchet MA Bachvaroff TR Tasumi S. Structural, functional, and evolutionary aspects of galectins in aquatic mollusks: from a sweet tooth to the Trojan horse. Fish Shellfish Immunol. (2015) 46:94–106. 10.1016/j.fsi.2015.05.012 25982395 PMC4509915

[62] Colnot C Sidhu SS Balmain N Poirier F. Uncoupling of chondrocyte death and vascular invasion in mouse galectin 3 null mutant bones. Dev Biol. (2001) 229:203–14. 10.1006/dbio.2000.9933 11133164

[63] Georgiadis V Stewart HJ Pollard HJ Tavsanoglu Y Prasad R Horwood J. Lack of galectin-1 results in defects in myoblast fusion and muscle regeneration. Dev Dyn. (2007) 236:1014–24. 10.1002/dvdy.21123 17366633

[64] Ahmed H Bianchet MA Amzel LM Hirabayashi J Kasai K Giga-Hama Y. Novel carbohydrate specificity of the 16-kDa galectin from *Caenorhabditis elegans*: binding to blood group precursor oligosaccharides (type 1, type 2, Talpha, and Tbeta) and gangliosides. Glycobiology. (2002) 12:451–61. 10.1093/glycob/cwf052 12145186

[65] Pace KE Lebestky T Hummel T Arnoux P Kwan K Baum LG. Characterization of a novel *Drosophila melanogaster* galectin. Expression in developing immune, neural, and muscle tissues. J Biol Chem. (2002) 277:13091–8. 10.1074/jbc.M112105200 11809773

[66] Vasta GR Ahmed H Du S Henrikson D. Galectins in teleost fish: Zebrafish (*Danio rerio*) as a model species to address their biological roles in development and innate immunity. Glycoconj J. (2004) 21:503–21. 10.1007/s10719-004-5541-7 15750792

[67] Nemoto-Sasaki Y Hayama K Ohya H Arata Y Kaneko MK Saitou N. *Caenorhabditis elegans* galectins LEC-1-LEC-11: structural features and sugar-binding properties. Biochim Biophys Acta. (2008) 1780:1131–42. 10.1016/j.bbagen.2008.07.003 18675319

[68] Feng C Nita-Lazar M Gonzalez-Montalban N Wang J Mancini J Ravindran C. Manipulating galectin expression in zebrafish (*Danio rerio*). Methods Mol Biol. (2015) 1207:327–41. 10.1007/978-1-4939-1396-1_22 25253151 PMC4600349

[69] Ahmed H Du SJ Vasta GR. Knockdown of a galectin-1-like protein in zebrafish (*Danio rerio*) causes defects in skeletal muscle development. Glycoconj J. (2009) 26:277–83. 10.1007/s10719-008-9178-9 18763034 PMC5345486

[70] Eastlake K Heywood WE Tracey-White D Aquino E Bliss E Vasta GR. Comparison of proteomic profiles in the zebrafish retina during experimental degeneration and regeneration. Sci Rep. (2017) 7:44601. 10.1038/srep44601 28300160 PMC5353638

[71] Lohr M Lensch M Andre S Kaltner H Siebert HC Smetana K Jr. Murine homodimeric adhesion/growth-regulatory galectins-1,−2 and−7: comparative profiling of gene/ promoter sequences by database mining, of expression by RT-PCR/immunohistochemistry and of contact sites for carbohydrate ligands by computational chemistry. Folia Biol. (2007) 53:109–28. 17706016 10.14712/fb2007053040109

[72] Guittaut M Charpentier S Normand T Dubois M Raimond J Legrand A Identification of an internal gene to the human Galectin-3 gene with two different overlapping reading frames that do not encode Galectin-3. J Biol Chem. (2001) 276:2652–7. 10.1074/jbc.M002523200 11160123

[73] Von Wolff M Wang X Gabius HJ Strowitzki T. Galectin fingerprinting in human endometrium and decidua during the menstrual cycle and in early gestation. Mol Hum Reprod. (2005) 11:189–94. 10.1093/molehr/gah144 15681515

[74] Freitag N Tirado-Gonzalez I Barrientos G Herse F Thijssen VL Weedon-Fekjaer SM. Interfering with Gal-1-mediated angiogenesis contributes to the pathogenesis of preeclampsia. Proc Natl Acad Sci USA. (2013) 110:11451–6. 10.1073/pnas.1303707110 23798433 PMC3710834

[75] Than NG Romero R Balogh A Karpati E Mastrolia SA Staretz-Chacham O. Galectins: double-edged swords in the cross-roads of pregnancy complications and female reproductive tract inflammation and neoplasia. J Pathol Transl Med. (2015) 49:181–208. 10.4132/jptm.2015.02.25 26018511 PMC4440931

[76] Mendez-Huergo SP Blidner AG Rabinovich GA. Galectins: emerging regulatory checkpoints linking tumor immunity and angiogenesis. Curr Opin Immunol. (2017) 45:8–15. 10.1016/j.coi.2016.12.003 28088061

[77] Tirado-Gonzalez I Freitag N Barrientos G Shaikly V Nagaeva O Strand M. Galectin-1 influences trophoblast immune evasion and emerges as a predictive factor for the outcome of pregnancy. Mol Hum Reprod. (2013) 19:43–53. 10.1093/molehr/gas043 23002109

[78] Fischer I Redel S Hofmann S Kuhn C Friese K Walzel H. Stimulation of syncytium formation *in vitro* in human trophoblast cells by galectin-1. Placenta. (2010) 31:825–32. 10.1016/j.placenta.2010.06.016 20656349

[79] Hutter S Morales-Prieto DM Andergassen U Tschakert L Kuhn C Hofmann S. Gal-1 silenced trophoblast tumor cells (BeWo) show decreased syncytium formation and different miRNA production compared to non-target silenced BeWo cells. Cell Adh Migr. (2016b) 10:28–38. 10.1080/19336918.2015.1089377 26418280 PMC4853045

[80] You JL Wang W Tang MY Ye YH Liu AX Zhu YM. A potential role of galectin-1 in promoting mouse trophoblast stem cell differentiation. Mol Cell Endocrinol. (2018) 470:228–39. 10.1016/j.mce.2017.11.003 29122660

[81] Jeschke U Mayr D Schiessl B Mylonas I Schulze S Kuhn C. Expression of galectin-1,−3 (gal-1, gal-3) and the Thomsen-Friedenreich (TF) antigen in normal, IUGR, preeclamptic and HELLP placentas. Placenta. (2007) 28:1165–73. 10.1016/j.placenta.2007.06.006 17664004

[82] Than NG Erez O Wildman DE Tarca AL Edwin SS Abbas A. Severe preeclampsia is characterized by increased placental expression of galectin-1. J Matern Fetal Neonatal Med. (2008b) 21:429–42. 10.1080/14767050802041961 18570123 PMC2775462

[83] Ramhorst RE Giribaldi L Fraccaroli L Toscano MA Stupirski JC Romero MD. Galectin-1 confers immune privilege to human trophoblast: implications in recurrent fetal loss. Glycobiology. (2012) 22:1374–86. 10.1093/glycob/cws104 22752006

[84] Schnabel A Blois SM Meint P Freitag N Ernst W Barrientos G. Elevated systemic galectin-1 levels characterize HELLP syndrome. J Reprod Immunol. (2016) 114:38–43. 10.1016/j.jri.2016.02.002 26956510

[85] Guardia CM Caramelo JJ Trujillo M Mendez-Huergo SP Radi R Estrin DA. Structural basis of redox-dependent modulation of galectin-1 dynamics and function. Glycobiology. (2014) 24:428–41. 10.1093/glycob/cwu008 24451991 PMC3976282

[86] Johannes L Jacob R Leffler H. Galectins at a glance. J Cell Sci. (2018) 131:jcs208884. 10.1242/jcs.208884 29717004

[87] Lakshminarayan R Wunder C Becken U Howes MT Benzing C Arumugam S. Galectin-3 drives glycosphingolipid-dependent biogenesis of clathrin-independent carriers. Nat Cell Biol. (2014) 16:595–606. 10.1038/ncb2970 24837829

[88] Advedissian T Proux-Gillardeaux V Nkosi R Peyret G Nguyen T Poirier F. E-cadherin dynamics is regulated by galectin-7 at epithelial cell surface. Sci Rep. (2017) 7:17086. 10.1038/s41598-017-17332-y 29213102 PMC5719072

[89] Vicovac L Jankovic M Cuperlovic M. Galectin-1 and−3 in cells of the first trimester placental bed. Hum Reprod. (1998) 13:730–5. 10.1093/humrep/13.3.730 9572443

[90] Kolundzic N Bojic-Trbojevic Z Kovacevic T Stefanoska I Kadoya T Vicovac L. Galectin-1 is part of human trophoblast invasion machinery–a functional study *in vitro*. PLoS ONE. (2011) 6:e28514. 10.1371/journal.pone.0028514 22174828 PMC3234277

[91] Chen Q Pang PC Cohen ME Longtine MS Schust DJ Haslam SM. Evidence for differential glycosylation of trophoblast cell types. Mol Cell Proteomics. (2016) 15:1857–66. 10.1074/mcp.M115.055798 26929217 PMC5083094

[92] Fischer C Sanchez-Ruderisch H Welzel M Wiedenmann B Sakai T Andre S Galectin-1 interacts with the α5β1 fibronectin receptor to restrict carcinoma cell growth via induction of p21 and p27. J Biol Chem. (2005) 280:37266–77. 10.1074/jbc.M411580200 16105842

[93] Sanchez-Ruderisch H Detjen KM Welzel M Andre S Fischer C Gabius HJ. Galectin-1 sensitizes carcinoma cells to anoikis via the fibronectin receptor α5β1-integrin. Cell Death Differ. (2011) 18:806–16. 10.1038/cdd.2010.148 21113146 PMC3131929

[94] Bojic-Trbojevic Z Krivokuca MJ Stefanoska I Kolundzic N Vilotic A Kadoya T. Integrin beta1 is bound to galectin-1 in human trophoblast. J Biochem. (2018) 163:39–50. 10.1093/jb/mvx061 28992109

[95] Bojic-Trbojevic Z Jovanovic Krivokuca M Kolundzic N Petronijevic M Vrzic-Petronijevic S Golubovic S. Galectin-1 binds mucin in human trophoblast. Histochem Cell Biol. (2014) 142:541–53. 10.1007/s00418-014-1229-7 24854997

[96] Shyu MK Chen CW Lin NY Liao WC Chen CH Lin CJ. MUC1 expression is elevated in severe preeclamptic placentas and suppresses trophoblast cell invasion via beta1-integrin signaling. J Clin Endocrinol Metab. (2011) 96:3759–67. 10.1210/jc.2011-1368 21917866

[97] Swanson BJ Mcdermott KM Singh PK Eggers JP Crocker PR Hollingsworth MA. MUC1 is a counter-receptor for myelin-associated glycoprotein (Siglec-4a) and their interaction contributes to adhesion in pancreatic cancer perineural invasion. Cancer Res. (2007) 67:10222–9. 10.1158/0008-5472.CAN-06-2483 17974963

[98] Shyu MK Lin MC Liu CH Fu YR Shih JC Lee CN. MUC1 expression is increased during human placental development and suppresses trophoblast-like cell invasion *in vitro*. Biol Reprod. (2008) 79:233–9. 10.1095/biolreprod.108.067629 18417712

[99] Hsieh SH Ying NW Wu MH Chiang WF Hsu CL Wong TY. Galectin-1, a novel ligand of neuropilin-1, activates VEGFR-2 signaling and modulates the migration of vascular endothelial cells. Oncogene. (2008) 27:3746–53. 10.1038/sj.onc.1211029 18223683

[100] Arad A Nammouz S Nov Y Ohel G Bejar J Vadasz Z. The expression of neuropilin-1 in human placentas from normal and preeclamptic pregnancies. Int J Gynecol Pathol. (2017) 36:42–9. 10.1097/PGP.0000000000000283 26937865

[101] Zhou Q Cummings RD. L-14 lectin recognition of laminin and its promotion of *in vitro* cell adhesion. Arch Biochem Biophys. (1993) 300:6–17. 10.1006/abbi.1993.1002 8380972

[102] Moiseeva EP Javed Q Spring EL De Bono DP. Galectin 1 is involved in vascular smooth muscle cell proliferation. Cardiovasc Res. (2000) 45:493–502. 10.1016/S0008-6363(99)00276-X 10728371

[103] Johnson GA Burghardt RC Bazer FW Spencer TE. Osteopontin: roles in implantation and placentation. Biol Reprod. (2003) 69:1458–71. 10.1095/biolreprod.103.020651 12890718

[104] Kim J Erikson DW Burghardt RC Spencer TE Wu G Bayless KJ. Secreted phosphoprotein 1 binds integrins to initiate multiple cell signaling pathways, including FRAP1/mTOR, to support attachment and force-generated migration of trophectoderm cells. Matrix Biol. (2010) 29:369–82. 10.1016/j.matbio.2010.04.001 20385232

[105] Koopman LA Kopcow HD Rybalov B Boyson JE Orange JS Schatz F. Human decidual natural killer cells are a unique NK cell subset with immunomodulatory potential. J Exp Med. (2003) 198:1201–12. 10.1084/jem.20030305 14568979 PMC2194228

[106] Aluvihare VR Kallikourdis M Betz AG. Regulatory T cells mediate maternal tolerance to the fetus. Nat Immunol. (2004) 5:266–71. 10.1038/ni1037 14758358

[107] Blois SM Ilarregui JM Tometten M Garcia M Orsal AS Cordo-Russo R. A pivotal role for galectin-1 in fetomaternal tolerance. Nat Med. (2007) 13:1450–7. 10.1038/nm1680 18026113

[108] Garin MI Chu CC Golshayan D Cernuda-Morollon E Wait R Lechler RI. Galectin-1: a key effector of regulation mediated by CD4+CD25+ T cells. Blood. (2007) 109:2058–65. 10.1182/blood-2006-04-016451 17110462

[109] Kopcow HD Rosetti F Leung Y Allan DS Kutok JL Strominger JL. T cell apoptosis at the maternal-fetal interface in early human pregnancy, involvement of galectin-1. Proc Natl Acad Sci USA. (2008) 105:18472–7. 10.1073/pnas.0809233105 19011096 PMC2587580

[110] Than NG Romero R Erez O Weckle A Tarca AL Hotra J. Emergence of hormonal and redox regulation of galectin-1 in placental mammals: implication in maternal-fetal immune tolerance. Proc Natl Acad Sci USA. (2008c) 105:15819–24. 10.1073/pnas.0807606105 18824694 PMC2556362

[111] Tamura M Sato D Nakajima M Saito M Sasaki T Tanaka T. Identification of galectin-2-mucin interaction and possible formation of a high molecular weight lattice. Biol Pharm Bull. (2017) 40:1789–95. 10.1248/bpb.b17-00221 28966253

[112] Loser K Sturm A Voskort M Kupas V Balkow S Auriemma M. Galectin-2 suppresses contact allergy by inducing apoptosis in activated CD8+ T cells. J Immunol. (2009) 182:5419–29. 10.4049/jimmunol.0802308 19380789

[113] Yildirim C Vogel DY Hollander MR Baggen JM Fontijn RD Nieuwenhuis S. Galectin-2 induces a proinflammatory, anti-arteriogenic phenotype in monocytes and macrophages. PLoS ONE. (2015) 10:e0124347. 10.1371/journal.pone.0124347 25884209 PMC4401781

[114] Hutter S Knabl J Andergassen U Hofmann S Kuhn C Mahner S. Placental expression patterns of galectin-1, galectin-2, galectin-3 and galectin-13 in cases of intrauterine growth restriction (IUGR). Int J Mol Sci. (2016a) 17:523. 10.3390/ijms17040523 27070577 PMC4848979

[115] Hutter S Martin N Von Schonfeldt V Messner J Kuhn C Hofmann S. Galectin 2 (gal-2) expression is downregulated on protein and mRNA level in placentas of preeclamptic (PE) patients. Placenta. (2015) 36:438–45. 10.1016/j.placenta.2015.01.198 25707742

[116] Unverdorben L Haufe T Santoso L Hofmann S Jeschke U Hutter S. Prototype and chimera-type galectins in placentas with spontaneous and recurrent miscarriages. Int J Mol Sci. (2016a) 17:644. 10.3390/ijms17050644 27136536 PMC4881470

[117] Sano H Hsu DK Apgar JR Yu L Sharma BB Kuwabara I. Critical role of galectin-3 in phagocytosis by macrophages. J Clin Invest. (2003) 112:389–97. 10.1172/JCI200317592 12897206 PMC166291

[118] Chen HY Liu FT Yang RY. Roles of galectin-3 in immune responses. Arch Immunol Ther Exp (Warsz). (2005) 53:497–504. 16407782

[119] Yang RY Hsu DK Liu FT. Expression of galectin-3 modulates T-cell growth and apoptosis. Proc Natl Acad Sci USA. (1996) 93:6737–42. 10.1073/pnas.93.13.6737 8692888 PMC39096

[120] Stillman BN Hsu DK Pang M Brewer CF Johnson P Liu FT. Galectin-3 and galectin-1 bind distinct cell surface glycoprotein receptors to induce T cell death. J Immunol. (2006) 176:778–89. 10.4049/jimmunol.176.2.778 16393961

[121] Maquoi E Van Den Brule FA Castronovo V Foidart JM. Changes in the distribution pattern of galectin-1 and galectin-3 in human placenta correlates with the differentiation pathways of trophoblasts. Placenta. (1997) 18:433–9. 10.1016/S0143-4004(97)80044-6 9250706

[122] Bozic M Petronijevic M Milenkovic S Atanackovic J Lazic J Vicovac L. Galectin-1 and galectin-3 in the trophoblast of the gestational trophoblastic disease. Placenta. (2004) 25:797–802. 10.1016/j.placenta.2004.03.006 15451194

[123] Yang H Lei C Zhang W. Expression of galectin-3 in mouse endometrium and its effect during embryo implantation. Reprod Biomed Online. (2012) 24:116–22. 10.1016/j.rbmo.2011.09.003 22119323

[124] Nangia-Makker P Honjo Y Sarvis R Akahani S Hogan V Pienta KJ. Galectin-3 induces endothelial cell morphogenesis and angiogenesis. Am J Pathol. (2000) 156:899–909. 10.1016/S0002-9440(10)64959-0 10702407 PMC1876842

[125] Evans J Yap J Gamage T Salamonsen L Dimitriadis E Menkhorst E. Galectin-7 is important for normal uterine repair following menstruation. Mol Hum Reprod. (2014) 20:787–98. 10.1093/molehr/gau032 24782449

[126] Menkhorst E Koga K Van Sinderen M Dimitriadis E. Galectin-7 serum levels are altered prior to the onset of pre-eclampsia. Placenta. (2014a) 35:281–5. 10.1016/j.placenta.2014.01.009 24534543

[127] Menkhorst EM Gamage T Cuman C Kaitu'u-Lino TJ Tong S Dimitriadis E. Galectin-7 acts as an adhesion molecule during implantation and increased expression is associated with miscarriage. Placenta. (2014b) 35:195–201. 10.1016/j.placenta.2014.01.004 24522232

[128] Cagli F Uysal G Oz L Akkaya H Aksoy H Ulku Karakilic E Serum galectin 7 is not useful to predict abortion in the first trimester. Fetal Pediatr Pathol. (2018) 37:1–6. 10.1080/15513815.2018.1453001 29693486

[129] Kolundzic N Bojic-Trbojevic Z Radojcic L Petronijevic M Vicovac L. Galectin-8 is expressed by villous and extravillous trophoblast of the human placenta. Placenta. (2011c) 32:909–11. 10.1016/j.placenta.2011.07.087 21862124

[130] Troncoso MF Ferragut F Bacigalupo ML Cardenas Delgado VM Nugnes LG Gentilini L. Galectin-8: a matricellular lectin with key roles in angiogenesis. Glycobiology. (2014) 24:907–14. 10.1093/glycob/cwu054 24939370

[131] Cattaneo V Tribulatti MV Carabelli J Carestia A Schattner M Campetella O. Galectin-8 elicits pro-inflammatory activities in the endothelium. Glycobiology. (2014) 24:966–73. 10.1093/glycob/cwu060 24957054

[132] Sampson JF Suryawanshi A Chen WS Rabinovich GA Panjwani N Galectin-8 promotes regulatory T-cell differentiation by modulating IL-2 and TGFbeta signaling. Immunol Cell Biol. (2016) 94:213–9. 10.1038/icb.2015.72 26282995 PMC4747822

[133] Carabelli J Quattrocchi V D'antuono A Zamorano P Tribulatti MV Campetella O. Galectin-8 activates dendritic cells and stimulates antigen-specific immune response elicitation. J Leukoc Biol. (2017) 102:1237–47. 10.1189/jlb.3A0816-357RR 28811319

[134] Pardo E Carcamo C Uribe-San Martin R Ciampi E Segovia-Miranda F Curkovic-Pena C. Galectin-8 as an immunosuppressor in experimental autoimmune encephalomyelitis and a target of human early prognostic antibodies in multiple sclerosis. PLoS ONE. (2017) 12:e0177472. 10.1371/journal.pone.0177472 28650992 PMC5484466

[135] Carabelli J Prato CA Sanmarco LM Aoki MP Campetella O Tribulatti MV Interleukin-6 signaling mediates Galectin-8 costimulatory activity of antigen-specific CD4 T cell response. Immunology. (2018) 155:379–86. 10.1111/imm.12980 29972692 PMC6187211

[136] Zick Y Eisenstein M Goren RA Hadari YR Levy Y Ronen D. Role of galectin-8 as a modulator of cell adhesion and cell growth. Glycoconj J. (2002) 19:517–26. 10.1023/B:GLYC.0000014081.55445.af 14758075

[137] Sanchez-Fueyo A Tian J Picarella D Domenig C Zheng XX Sabatos CA. Tim-3 inhibits T helper type 1-mediated auto- and alloimmune responses and promotes immunological tolerance. Nat Immunol. (2003) 4:1093–101. 10.1038/ni987 14556005

[138] Bi S Hong PW Lee B Baum LG. Galectin-9 binding to cell surface protein disulfide isomerase regulates the redox environment to enhance T-cell migration and HIV entry. Proc Natl Acad Sci USA. (2011) 108:10650–5. 10.1073/pnas.1017954108 21670307 PMC3127870

[139] Wu C Thalhamer T Franca RF Xiao S Wang C Hotta C. Galectin-9-CD44 interaction enhances stability and function of adaptive regulatory T cells. Immunity. (2014) 41:270–82. 10.1016/j.immuni.2014.06.011 25065622 PMC4219323

[140] Popovici RM Krause MS Germeyer A Strowitzki T Von Wolff M. Galectin-9: a new endometrial epithelial marker for the mid- and late-secretory and decidual phases in humans. J Clin Endocrinol Metab. (2005) 90:6170–6. 10.1210/jc.2004-2529 16105962

[141] Shimizu Y Kabir-Salmani M Azadbakht M Sugihara K Sakai K Iwashita M. Expression and localization of galectin-9 in the human uterodome. Endocr J. (2008) 55:879–87. 10.1507/endocrj.K08E-111 18506087

[142] Thijssen VL Hulsmans S Griffioen AW. The galectin profile of the endothelium: altered expression and localization in activated and tumor endothelial cells. Am J Pathol. (2008) 172:545–53. 10.2353/ajpath.2008.070938 18202194 PMC2312370

[143] Heusschen R Freitag N Tirado-Gonzalez I Barrientos G Moschansky P Munoz-Fernandez R. Profiling Lgals9 splice variant expression at the fetal-maternal interface: implications in normal and pathological human pregnancy. Biol Reprod. (2013) 88:22. 10.1095/biolreprod.112.105460 23242525

[144] Zhang F Zheng M Qu Y Li J Ji J Feng B. Different roles of galectin-9 isoforms in modulating E-selectin expression and adhesion function in LoVo colon carcinoma cells. Mol Biol Rep. (2009) 36:823–30. 10.1007/s11033-008-9251-2 18401566

[145] Li Y Feng J Geng S Geng S Wei H Chen G. The N- and C-terminal carbohydrate recognition domains of galectin-9 contribute differently to its multiple functions in innate immunity and adaptive immunity. Mol Immunol. (2011) 48:670–7. 10.1016/j.molimm.2010.11.011 21146220

[146] Meggyes M Miko E Polgar B Bogar B Farkas B Illes Z. Peripheral blood TIM-3 positive NK and CD8+ T cells throughout pregnancy: TIM-3/galectin-9 interaction and its possible role during pregnancy. PLoS ONE. (2014) 9:e92371. 10.1371/journal.pone.0092371 24651720 PMC3961322

[147] Enninga EA Nevala WK Creedon DJ Markovic SN Holtan SG. Fetal sex-based differences in maternal hormones, angiogenic factors, and immune mediators during pregnancy and the postpartum period. Am J Reprod Immunol. (2015) 73:251–62. 10.1111/aji.12303 25091957 PMC4317383

[148] Enninga EAL Harrington SM Creedon DJ Ruano R Markovic SN Dong H. Immune checkpoint molecules soluble program death ligand 1 and galectin-9 are increased in pregnancy. Am J Reprod Immunol. (2018) 79:e12795. 10.1111/aji.12795 29205636 PMC5814874

[149] Li Y Zhang J Zhang D Hong X Tao Y Wang S. Tim-3 signaling in peripheral NK cells promotes maternal-fetal immune tolerance and alleviates pregnancy loss. Sci Signal. (2017) 10:eaah4323. 10.1126/scisignal.aah4323 28951537

[150] He M Jiang M Zhou Y Li F Yang M Fan Y. Impaired Gal-9 dysregulates the PBMC-induced Th1/Th2 imbalance in abortion-prone matings. J Immunol Res. (2018) 2018:9517842. 10.1155/2018/9517842 29651447 PMC5831994

[151] Lajko A Meggyes M Polgar B Szereday L. The immunological effect of Galectin-9/TIM-3 pathway after low dose Mifepristone treatment in mice at 14.5 day of pregnancy. PLoS ONE. (2018) 13:e0194870. 10.1371/journal.pone.0194870 29566059 PMC5864070

[152] Li ZH Wang LL Liu H Muyayalo KP Huang XB Mor G. Galectin-9 alleviates LPS-induced preeclampsia-like impairment in rats via switching decidual macrophage polarization to M2 subtype. Front Immunol. (2018) 9:3142. 10.3389/fimmu.2018.03142 30687334 PMC6335255

[153] Abedin MJ Kashio Y Seki M Nakamura K Hirashima M. Potential roles of galectins in myeloid differentiation into three different lineages. J Leukoc Biol. (2003) 73:650–6. 10.1189/jlb.0402163 12714580

[154] Kubach J Lutter P Bopp T Stoll S Becker C Huter E. Human CD4+CD25+ regulatory T cells: proteome analysis identifies galectin-10 as a novel marker essential for their anergy and suppressive function. Blood. (2007) 110:1550–8. 10.1182/blood-2007-01-069229 17502455

[155] Unverdorben L Jeschke U Santoso L Hofmann S Kuhn C Arck P. Comparative analyses on expression of galectins1-4, 7-10 and 12 in first trimester placenta, decidua and isolated trophoblast cells *in vitro*. Histol Histopathol. (2016b) 31:1095–111. 10.14670/HH-11-739. 26901464

[156] Than NG Romero R Kim CJ Mcgowen MR Papp Z Wildman DE. Galectins: guardians of eutherian pregnancy at the maternal-fetal interface. Trends Endocrinol Metab. (2012) 23:23–31. 10.1016/j.tem.2011.09.003 22036528 PMC3640805

[157] Bohn H Kraus W Winckler W. Purification and characterization of two new soluble placental tissue proteins (PP13 and PP17). Oncodev Biol Med. (1983) 4:343–50. 6856484

[158] Than NG Sumegi B Than GN Berente Z Bohn H Isolation and sequence analysis of a cDNA encoding human placental tissue protein 13 (PP13), a new lysophospholipase, homolog of human eosinophil Charcot-Leyden Crystal protein. Placenta. (1999) 20:703–10. 10.1053/plac.1999.0436 10527825

[159] Than NG Pick E Bellyei S Szigeti A Burger O Berente Z. Functional analyses of placental protein 13/galectin-13. Eur J Biochem. (2004) 271:1065–78. 10.1111/j.1432-1033.2004.04004.x 15009185

[160] Burger O Pick E Zwickel J Klayman M Meiri H Slotky R. Placental protein 13 (PP-13): effects on cultured trophoblasts, and its detection in human body fluids in normal and pathological pregnancies. Placenta. (2004) 25:608–22. 10.1016/j.placenta.2003.12.009 15193867

[161] Huppertz B Sammar M Chefetz I Neumaier-Wagner P Bartz C Meiri H. Longitudinal determination of serum placental protein 13 during development of preeclampsia. Fetal Diagn Ther. (2008) 24:230–6. 10.1159/000151344 18753763

[162] Sammar M Dragovic R Meiri H Vatish M Sharabi-Nov A Sargent I. Reduced placental protein 13 (PP13) in placental derived syncytiotrophoblast extracellular vesicles in preeclampsia - a novel tool to study the impaired cargo transmission of the placenta to the maternal organs. Placenta. (2018) 66:17–25. 10.1016/j.placenta.2018.04.013 29884298

[163] Boronkai A Bellyei S Szigeti A Pozsgai E Bognar Z Sumegi B. Potentiation of paclitaxel-induced apoptosis by galectin-13 overexpression via activation of Ask-1-p38-MAP kinase and JNK/SAPK pathways and suppression of Akt and ERK1/2 activation in U-937 human macrophage cells. Eur J Cell Biol. (2009) 88:753–63. 10.1016/j.ejcb.2009.07.005 19717209

[164] Gizurarson S Huppertz B Osol G Skarphedinsson JO Mandala M Meiri H. Effects of placental protein 13 on the cardiovascular system in gravid and non-gravid rodents. Fetal Diagn Ther. (2013) 33:257–64. 10.1159/000345964 23406577

[165] Than NG Balogh A Romero R Karpati E Erez O Szilagyi A. Placental protein 13 (PP13) - a placental immunoregulatory galectin protecting pregnancy. Front Immunol. (2014a) 5:348. 10.3389/fimmu.2014.00348 25191322 PMC4138504

[166] Drobnjak T Gizurarson S Gokina NI Meiri H Mandala M Huppertz B. Placental protein 13 (PP13)-induced vasodilation of resistance arteries from pregnant and nonpregnant rats occurs via endothelial-signaling pathways. Hypertens Pregn. (2017) 36:186–95. 10.1080/10641955.2017.1295052 28524718

[167] Than NG Abdul Rahman O Magenheim R Nagy B Fule T Hargitai B. Placental protein 13 (galectin-13) has decreased placental expression but increased shedding and maternal serum concentrations in patients presenting with preterm pre-eclampsia and HELLP syndrome. Virchows Arch. (2008a) 453:387–400. 10.1007/s00428-008-0658-x 18791734 PMC2775473

[168] Spencer K Cowans NJ Chefetz I Tal J Kuhnreich I Meiri H. Second-trimester uterine artery Doppler pulsatility index and maternal serum PP13 as markers of pre-eclampsia. Prenat Diagn. (2007) 27:258–63. 10.1002/pd.1664 17278173

[169] Huppertz B. Biology of preeclampsia: combined actions of angiogenic factors, their receptors and placental proteins. Biochim Biophys Acta Mol Basis Dis. (2018). S0925–4439(18)30486-1. [Epub ahead of print]. 10.1016/j.bbadis.2018.11.024 30553017

[170] Unverdorben L Huttenbrenner R Knabl J Jeschke U Hutter S. Galectin-13/PP-13 expression in term placentas of gestational diabetes mellitus pregnancies. Placenta. (2015) 36:191–8. 10.1016/j.placenta.2014.11.019 25499680

[171] Zhao B Han X Meng Q Luo Q. Early second trimester maternal serum markers in the prediction of gestational diabetes mellitus. J Diabetes Investig. (2018) 9:967–74. 10.1111/jdi.12798 29288571 PMC6031488

[172] Su J Wang Y Si Y Gao J Song C Cui L Galectin-13, a different prototype galectin, does not bind beta-galacto-sides and forms dimers via intermolecular disulfide bridges between Cys-136 and Cys-138. Sci Rep. (2018) 8:980 10.1038/s41598-018-19465-0 29343868 PMC5772480

[173] Than NG Romero R Meiri H Erez O Xu Y Tarquini F. PP13, maternal ABO blood groups and the risk assessment of pregnancy complications. PLoS ONE. (2011) 6:e21564. 10.1371/journal.pone.0021564 21799738 PMC3143125

[174] Dent J Mcgovern PT Hancock JL. Immunological implications of ultrastructural studies of goat X sheep hybrid placentae. Nature. (1971) 231:116–7. 10.1038/231116a0 4930089

[175] Tomiie M Isaka S Miyoshi E Taniguchi N Kimura T Ogita K. Elevated expression of N-acetylglucosaminyltransferase V in first trimester human placenta. Biochem Biophys Res Commun. (2005) 330:999–1004. 10.1016/j.bbrc.2005.02.186 15809094

[176] Yamamoto E Ino K Miyoshi E Inamori K Abe A Sumigama S. N-acetylglucosaminyltransferase V regulates extravillous trophoblast invasion through glycosylation of alpha5beta1 integrin. Endocrinology. (2009) 150:990–9. 10.1210/en.2008-1005 18845630

[177] Ji L Brkic J Liu M Fu G Peng C Wang YL. Placental trophoblast cell differentiation: physiological regulation and pathological relevance to preeclampsia. Mol Aspects Med. (2013) 34:981–1023. 10.1016/j.mam.2012.12.008 23276825

[178] Jansen BC Bondt A Reiding KR Lonardi E De Jong CJ Falck D. Pregnancy-associated serum N-glycome changes studied by high-throughput MALDI-TOF-MS. Sci Rep. (2016) 6:23296. 10.1038/srep23296 27075729 PMC4831011

[179] Varki A. Biological roles of oligosaccharides: all of the theories are correct. Glycobiology. (1993) 3:97–130. 10.1093/glycob/3.2.97 8490246 PMC7108619

[180] Acheson A Sunshine JL Rutishauser U. NCAM polysialic acid can regulate both cell-cell and cell-substrate interactions. J Cell Biol. (1991) 114:143–53. 10.1083/jcb.114.1.143 2050739 PMC2289064

[181] Lauc G Zoldos V. Protein glycosylation–an evolutionary crossroad between genes and environment. Mol Biosyst. (2010) 6:2373–9. 10.1039/c0mb00067a 20957246

[182] Kaszuba K Grzybek M Orlowski A Danne R Rog T Simons K. N-Glycosylation as determinant of epidermal growth factor receptor conformation in membranes. Proc Natl Acad Sci USA. (2015) 112:4334–9. 10.1073/pnas.1503262112 25805821 PMC4394299

[183] Jones CJ Carter AM Aplin JD Enders AC. Glycosylation at the fetomaternal interface in hemomonochorial placentae from five widely separated species of mammal: is there evidence for convergent evolution? Cells Tissues Organs. (2007) 185:269–84. 10.1159/000102175 17587801

[184] Mcmaster M Zhou Y Shorter S Kapasi K Geraghty D Lim KH. HLA-G isoforms produced by placental cytotrophoblasts and found in amniotic fluid are due to unusual glycosylation. J Immunol. (1998) 160:5922–8. 9637505

[185] Moss L Prakobphol A Wiedmann TW Fisher SJ Damsky CH. Glycosylation of human trophoblast integrins is stage and cell-type specific. Glycobiology. (1994) 4:567–75. 10.1093/glycob/4.5.567 7881170

[186] Gleeson LM Chakraborty C Mckinnon T Lala PK. Insulin-like growth factor-binding protein 1 stimulates human trophoblast migration by signaling through alpha 5 beta 1 integrin via mitogen-activated protein Kinase pathway. J Clin Endocrinol Metab. (2001) 86:2484–93. 10.1210/jc.86.6.2484 11397844

[187] Jovanovic M Stefanoska I Radojcic L Vicovac L. Interleukin-8 (CXCL8) stimulates trophoblast cell migration and invasion by increasing levels of matrix metalloproteinase (MMP)2 and MMP9 and integrins alpha5 and beta1. Reproduction. (2010) 139:789–98. 10.1530/REP-09-0341 20133364

[188] Li CM Hou L Zhang H Zhang WY CCL17 induces trophoblast migration and invasion by regulating matrix metalloproteinase and integrin expression in human first-trimester placenta. Reprod Sci. (2014) 1933719113519170 10.1177/1933719113519170 [Epub ahead of print]. 24401476

[189] Liao WC Liu CH Chen CH Hsu WM Liao YY Chang HM. beta-1,4-Galactosyltransferase III suppresses extravillous trophoblast invasion through modifying beta1-integrin glycosylation. Placenta. (2015) 36:357–64. 10.1016/j.placenta.2015.01.008 25659296

[190] Furmento VA Marino J Blank VC Cayrol MF Cremaschi GA Aguilar RC. Granulocyte colony-stimulating factor (G-CSF) upregulates beta1 integrin and increases migration of human trophoblast Swan 71 cells via PI3K and MAPK activation. Exp Cell Res. (2016) 342:125–34. 10.1016/j.yexcr.2016.03.005 26992288 PMC5338037

[191] Zhang M Wang M Gao R Liu X Chen X Geng Y. Altered beta1,6-GlcNAc and bisecting GlcNAc-branched N-glycan on integrin beta1 are associated with early spontaneous miscarriage in humans. Hum Reprod. (2015) 30:2064–75. 10.1093/humrep/dev153 26109616

[192] Deng Q Chen Y Yin N Shan N Luo X Yuan Y. The role of MGAT5 in human umbilical vein endothelial cells. Reprod Sci. (2017) 24:313–23. 10.1177/1933719116655602 27334383

[193] Tannetta D Masliukaite I Vatish M Redman C Sargent I. Update of syncytiotrophoblast derived extracellular vesicles in normal pregnancy and preeclampsia. J Reprod Immunol. (2017) 119:98–106. 10.1016/j.jri.2016.08.008 27613663

[194] Toscano MA Bianco GA Ilarregui JM Croci DO Correale J Hernandez JD. Differential glycosylation of TH1, TH2 and TH-17 effector cells selectively regulates susceptibility to cell death. Nat Immunol. (2007) 8:825–34. 10.1038/ni1482 17589510

[195] Cerliani JP Blidner AG Toscano MA Croci DO Rabinovich GA. Translating the 'Sugar Code' into immune and vascular signaling programs. Trends Biochem Sci. (2017) 42:255–73. 10.1016/j.tibs.2016.11.003 27986367

[196] Marini M Bonaccini L Thyrion GD Vichi D Parretti E Sgambati E. Distribution of sugar residues in human placentas from pregnancies complicated by hypertensive disorders. Acta Histochem. (2011) 113:815–25. 10.1016/j.acthis.2010.12.001 21774970

[197] Di Lella S Sundblad V Cerliani JP Guardia CM Estrin DA Vasta GR. When galectins recognize glycans: from biochemistry to physiology and back again. Biochemistry. (2011) 50:7842–57. 10.1021/bi201121m 21848324 PMC3429939

